# Some Structural Aspects of Language Are More Stable than Others: A Comparison of Seven Methods

**DOI:** 10.1371/journal.pone.0055009

**Published:** 2013-01-28

**Authors:** Dan Dediu, Michael Cysouw

**Affiliations:** 1 Language and Genetics Department, Max Planck Institute for Psycholinguistics, Nijmegen, The Netherlands; 2 Donders Institute for Brain, Cognition and Behaviour, Nijmegen, The Netherlands; 3 Forschungszentrum Deutscher Sprachatlas, Philipps Universität Marburg, Marburg, Germany; New York State Museum, United States of America

## Abstract

Understanding the patterns and causes of differential structural stability is an area of major interest for the study of language change and evolution. It is still debated whether structural features have intrinsic stabilities across language families and geographic areas, or if the processes governing their rate of change are completely dependent upon the specific context of a given language or language family. We conducted an extensive literature review and selected seven different approaches to conceptualising and estimating the stability of structural linguistic features, aiming at comparing them using the same dataset, the *World Atlas of Language Structures*. We found that, despite profound conceptual and empirical differences between these methods, they tend to agree in classifying some structural linguistic features as being more stable than others. This suggests that there are intrinsic properties of such structural features influencing their stability across methods, language families and geographic areas. This finding is a major step towards understanding the nature of structural linguistic features and their interaction with idiosyncratic, lineage- and area-specific factors during language change and evolution.

## Introduction

Languages always change no matter how vocal prescriptivists are and how strongly the rules of “good” language are enforced [1]. However, different languages – and even different aspects of a single language – change in different manners and at different rates. For example, Icelandic is notoriously conservative among the Germanic languages [2], while English has relatively suddenly lost most of its morphological case marking system inherited from Old English. Understanding the patterns and causes of differential structural stability is an area of major interest for the study of language change and evolution. We will first briefly discuss the notion of stability in the context of molecular biology, before turning to previous research on stability in linguistics.

### Stability in Biology

This situation is similar to evolutionary biology, where stability (and its complement, the rate of evolution) are complex outcomes of multiple factors, including universal and lineage-specific components. Neutral genetic markers evolve at a constant rate dictated by mutation rate [3] resulting in a molecular clock, while nearly neutral markers evolve at a rate determined by mutation and population size [4], reflecting the balance between mutation (production of novelty) and genetic drift (purging variation from the population). However, this is complicated by the non-constancy of mutation rates across the genome, across species and time, being influenced by, among others, the local DNA context, metabolism, life history parameters, age, gender and environmental stress [5], [6]. The various types of natural selection add a supplementary level of complexity. For example, purifying selection will tend to resist change, while positive selection will increase the rates of evolution [7].

Thus, there are highly conserved genes, such as those coding for ribosomal RNA present across the whole of cellular life and covering at least 3.5 billion years [8] or the *Pax6* gene (a master gene controlling the cascade leading to eye development) so well conserved that the mouse gene induces eye formation in the fruit fly [9], while the fruit fly homologue genes *eyeless* and *twin of eyeless* induce the formation of several eye structures in the frog embryos [10]. At the other end of the spectrum, there are genes which evolve extremely fast, such as some involved in the immune system [11] or male reproductive biology [12], [13], where strong and dynamic natural or sexual selective pressures are acting. Interestingly, there are also stretches of DNA which, despite being very stable in general, have changed a lot in a given lineage, such as the so-called human accelerated regions (HARs; [14], [15]) which have changed dramatically in the lineage leading to us. Several genes involved in microcephaly [16], such as *ASPM*, *Microcephalin* and *SHH*, show faster evolution in primates and especially in the lineage leading to humans, suggesting that they might have been partly responsible for the evolution of increased human brain size. *FOXP2*, a gene involved in developmental verbal dyspraxia [17] is one of the most conserved genes within vertebrates [18], but modern humans and Neandertals carry a specific variant which differs at 2 positions from the chimpanzee [18], [19]. A proposed explanation for these differences in patterns of stability among genes is represented by the *extended complexity hypothesis* [20], suggesting that genes that are involved in complex and extensive interactions, and whose products participate in informational processes (transcription, translation and related aspects) and other complex functions, tend to change more slowly.

### Stability of Vocabulary

In the case of language, recent work [21]–[23] shows that not all concepts in a list of basic vocabulary – i.e. a standardised list of concepts selected for their universality, the best-known being the Swadesh 100 and 200-words lists [24] – are equally stable. For example, concepts such as “two”, “who”, “tongue”, “night”, “one” and “to die” seem to be extremely stable in the Indo-European language family, showing at most 1 cognate replacement per 10,000 years of language change (depending on the assumed age of the family), while the most unstable meanings, such as “dirty”, “to turn”, “to stab” and “guts”, show up to 9 such replacements during the same period [22]. Moreover, the stability of these concepts has a relatively strong universal component, in the sense that their relative stabilities tend to be conserved across several different language families [22], [23], [25], [26]. An important explanatory factor seems to be the frequency of use of these concepts, with the more frequently used tending to be more stable [22]. Thus, it seems that certain concepts have a set of properties, including their frequency of use, which tend to make them resilient against lexical replacement across language families, time and space, the most stable showing a fidelity comparable to that of genetic systems [23].

### Stability of Structural Linguistic Features

The properties and patterns of stability of *structural* aspects of language, such as the order of subject and verb or the number of consonants in a language, are less well understood. Some authors [27]–[29] suggest that the distributional properties of structural features might inform us about deeper historical relationships than are accessible through the standard comparative method of historical linguistics [30], [31], and they seem, at least in some cases, more resistant to admixture than human genes [32].

In contrast (and in agreement with widespread assumptions in historical linguistics), recent work [26] compared the historical signal and phylogenetic stability of the basic vocabulary to that of structural features in the Indo-European and the Austronesian language families and found that in both families the vocabulary data fitted the comparative-method established family trees much better than the structural data. This suggests that structural features evolve much faster and/or are more influenced by contact phenomena [33] than basic vocabulary. Moreover, the rates of evolution were roughly similar for vocabulary and structural data in both families but the structural features stabilities’ (in contrast to the vocabulary) show very weak correlations across these language families, leading the authors to conclude that they “do not support the existence of a set of universally stable typological features” (p.6). Likewise, other recent work [34] suggests that structural properties are language family-specific, although this work was not directly aimed at studying the stability of structural features but to understanding the regularities governing the temporal dynamics (i.e. correlated evolution) of various aspects of word order. Both these studies suffer from a limited coverage of different language families, potentially undermining the generality of their findings. By considering more language families and structural features, recent work involving the first author [35], [36] seems to reconcile these two views by finding that there is an important universal, cross-language family component to the stability of structural features, but that there is also a non-negligible amount of variation among families. The view that multiple factors, such as universal tendencies, vertical and horizontal processes are requried to explain linguistic diversity has been suggested before (e.g., [27], [37]), but more empirical work using large databases and modern quantitative methods is required for a thorough understanding of this complex interplay [36].

There is currently a vigorous debate concerning the stability of the structural properties of languages discussing whether (i) there are universal, cross-language family tendencies in that some features are more stable than others, or (ii) the stability of a feature is entirely a language family-idiosyncratic property. The first view points to possible universal biases acting on structural features which influence their stability. These biases can be due to communicative pressures, or to extra-linguistic factors (such as neuro-physiologic, cognitive, articulatory and perceptive constraints), or to factors related to the linguistic system itself. As a result, some features might play a more central role in shaping the structural system of a language (alike to the model of the extended complexity hypothesis [20] in biology). The second view instead suggests that “historical accidents” (or “driving factors” in [38]) specific to individual language families are the major determinants of structural change. Of course, there is the third possibility [35], [36], namely that these two views are not mutually exclusive but complementary.

### Summary of Paper

The present paper represents an *empirical* approach to the issues surrounding the stability of structural features. Here, we compare seven different published methods of estimating the stability of structural linguistic features, in order to quantify their overlap and differences. These seven methods propose different definitions of the concept of structural feature stability and different estimation techniques of these stabilities, while using the same large database of language families and features, namely the *World Atlas of Language Structures* (WALS) [39]. Reviewing the various methods, we found that the seemingly simple concept of structural stability hides an irreducible complexity, mainly due to the prevalence and importance of horizontal processes in language change [40] and the manner in which various proposals acknowledge and quantify them. We believe that conceptual clarity about structural stability is a necessary step in any discussion concerning language change and evolution, and our empirical approach complements theoretical frameworks such as Nichols’ [41].

Most importantly, we found that, despite this variety of conceptualizations and methodological approaches, there is an important agreement between these different stability estimates. This strongly suggests the existence of a universal, cross-language family component, probably due to intrinsic properties of the structural features above and beyond the particular constraints of the specific language families and areas. However, this universal component does not explain the whole range of variation in structural stability, showing that there are also language family-specific factors at work. These findigs might help better understand the interplay between the various “competing forces” and the relationship between different types of stability in particular language families and areas and for particular structural features [38], [41]. We hope that these findings will open the door to a research program aiming at understanding the nature and exact mixture of universal and idiosyncratic components governing structural language change and evolution.

## Materials and Methods

We conducted an extensive literature survey in order to identify and compare different proposals about the concept of *stability* as applied to structural features of language. Given the differences between proposals that our survey revealed, only a very general gist of this concept (or rather of its opposite, *instability*) can be formulated, namely as the easiness with which features change value across time, under the influence of various processes. To ensure comparability and objectivity, we defined several criteria the proposals must meet in order to be considered:

1. they must be described in *published* form or in *publicly available* drafts designed for publication;
2. they must use a *concept of stability* fitting our general gist;
3. they must be *quantifiable*, *objective* and *repeatable*;
4. they must deal with *many structural features*, preferably using the WALS or equivalent datasets, allowing thus the comparison with other methods;
5. they must produce estimates across *many language families*, preferably using the WALS, the Ethnologue or equivalent classifications, allowing broad comparability and cross-checking; and
6. they must produce at least a *rank of feature stabilities* from the most stable to the most unstable.

We found seven methods that meet our criteria and we briefly describe them in the alphabetic order of the first author. All these methods used the structural features, their values and the language families as given by WALS [39], except for the method described in [35] which also used the classification given in the Ethnologue [42].

### Cysouw, Albu & Dress (2008): the Consistency with Overall Patterns

These authors [43] develop a very original take on the issue of stability, in that their primary interest is in identifying *consistent* structural features. Such features “are most indicative of the overall structure of a language […] of the typological profile, or ‘genius’ of a language” (p.263) and are identified by comparing their distributional properties with the “averaged” distribution of many features. The fundamental insight is to compute the typological (structural) distances between languages relative to each structural feature and to quantify how accurately the typological distance given by any single feature reflects the overall typological distance given by all features considered simultaneously.

More specifically, the authors start by defining the typological distance 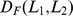 between a pair of languages 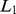 and 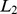 relative to a feature 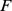 as being 0 if both languages share the same attested feature value, 2 if they have attested but different values, and 1 if the feature value for at least one language is unattested (missing data). This is extended to a set of features 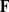 by taking the average of 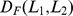 for all features 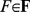 which have attested values in both languages. This leads naturally to a set of distance matrices between all pairs of languages, first, one matrix per feature, 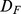, and, second, an overall distance matrix 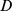 computed for all considered features. The features 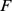 for which 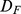 is more similar to 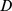 are defined as more consistent. The authors propose three ways to quantify this fit between 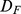 and 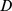:

- *Mantel’s congruence test*, denoted in the following by **CM**, is based on Mantel’s proposal [44] to compute the similarity between the two matrices 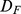 and 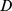 as the proportion of matrices 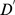 (obtained by randomly permuting 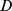
’s rows) which have a higher correlation with 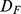. In effect, this method uses the inverse of the *p*-value derived from the permutations test as the measure of consistency;
- the *coherence* method (**CC**) is based on the “excess” of two languages 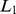 and 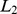 relative to a third language 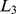 given a distance matrix 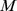, denoted 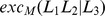 and computed as 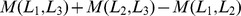 averaged across all languages for the feature 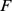, versus the overall distances 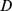. The excess 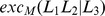 measures the extra distance between languages 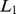 and 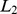 when taking a detour through language 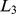. With these, the coherence of feature 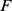 is the ratio of the excess given 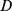 to the excess given 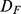 averaged across all possible triplets of languages, 
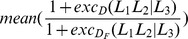
;
- the *rank* method (**CR**) is based on the rank of a language 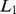 relative to another language 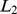 defined as the number of languages whose distance to 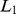 is smaller than the distance of 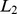 to 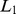, 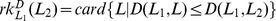 With these, the rank matrix between languages is defined as 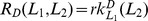, and the coherence of a feature 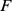 is computed based on the average ranks of languages sharing the same feature values, 
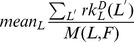
, where 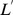 have the same value for 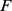 as 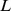, and 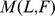 is the smallest possible value of the sum in the numerator. Thus, the method quantifies the ranks of languages that share the feature value with 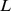 averaged across all languages 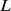.

Thus, there are three different ways of measuring the consistency of a structural feature with the overall pattern of all features, each quantifying it in a different manner. Given the complexity of these methods, we urge the interested readers to consult the original paper [43] for a better understanding of their details.

The authors report that (i) these quantifications tend to give comparable results across different datasets (with **CC** being the most resilient), that (ii) their sensitivity to the amount of missing data varies dramatically between methods (with **CR** being the least sensitive), that (iii) they seem relatively unaffected by the distribution of feature values and, surprisingly, that (iv) they do not inter-correlate very well (except for **CC** and **CR**, for which 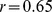, 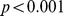).

Finally, and importantly for the present paper, the authors tested the relationship between consistency and genealogical stability by comparing the distances between related and unrelated languages using sets of the most consistent 25%, 50% and 75% features, and found that related languages had significantly lower distances than unrelated languages. This suggests that the most consistent features might be genealogically stable as well, in the sense that they do distinguish between related and unrelated languages, their values tending to be inherited. If such consistent features do indeed reflect the “typological profile” of the languages and if we assume that such profiles tend to be vertically transmitted, then consistent features will also be stable in the genealogical sense, being inherited from ancestor to daughter languages.

### Dediu (2011): the Phylogenetic Rates of Evolution

The approach from [35] (denoted in the following as **D**) estimates the stability of structural features from a Bayesian phylogenetic perspective [45], [46]. More precisely, for a given language family, the observed values of the structural features within this family’s languages together with the tree representing the genealogical relationships between these languages are used to infer the rates at which the structural features have changed in this family. As is specific to Bayesian methods in general [47], [48], this results in a posterior distribution of such rates giving the posterior probability that a particular structural feature changes at a particular rate in this family.

The author uses two methods for estimating the rates of structural change: (i) a method estimating the probability of a transition between two feature values during an infinitesimally short time, implemented in MrBayes 3 [49], and (ii) another method related to maximum parsimony [50] estimating the minimum number of changes required to produce the observed feature values starting from the inferred root value, implemented in BayesLang [35]. It must be highlighted that these phylogenetic methods are agnostic in what concerns the cause for these structural changes: they could include “spontaneous mutation”, borrowing, language shift and various forms of selective pressures acting on language (“driving factors”) such as cognitive, perceptive or articulatory biases. Therefore, non-vertical processes are treated as just another cause of structural change and reflected in the posterior distribution of rates [36].

In order to control for the influence of particular historical classifications, the author used the classifications given by WALS [39] and Ethnologue [42], later [36] extending this to a collection of more accepted classifications [51]. Given that absolute rates of change cannot be directly compared across language families (as it would require the absolute dating of the root proto-languages), they were converted to ranks with features ordered from the most to the least stable. This reduction in measurement level to ordinal ensures comparability not only across family trees but also across different methods. Most importantly, all combinations of the two methods of stability estimation and the three classifications produced highly similar estimates for the rates of structural change [35], [36].

### Maslova (2002, 2004): Estimating Transition Probabilities

Elena Maslova [52]–[55] proposed a method to estimate the transition probabilities between the values of a given structural feature using pairs of closely related languages. Basically (for a complete exposition of Maslova’s method see [56] and especially **Methods S1** here, where we also give the R code [**Script S1**] used to implement the method using the WALS data [**Dataset S1**] ), for a binary structural feature 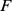 which can take values 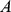 and 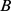, there are four possible transitions in a given time period for a given language: 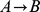 with probability 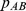, 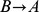 with probability 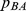, 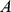 does not change with probability 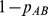, and 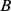 does not change with probability 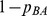. With these, the stability of feature 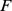 is
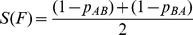



To estimate 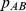 and 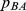, we need to sample pairs of related languages and compute the divergence rate, 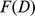, defined as the proportion of such pairs differing for feature 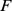. If we denote the frequency of languages with value 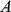 for feature 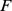 as 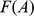, Maslova derives the following equation (see the Supporting Online Information):
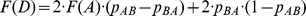
allowing the estimation of 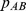 and 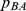 from at least two such samples.

This method is based on the same fundamental insights [56] as the fully phylogenetic methods discussed above [35], but it uses a much simpler statistical approach and requires stronger assumptions concerning the relationships between the pairs of closely related languages. We have implemented this method in R [57] and estimated the stability of the structural features in WALS [39] using WALS’ genera (the intermediate level between individual languages and language families, such as *Germanic* and *Romance* within *Indo-European*) to provide the sets of closely related languages (these estimates are denoted in the following as **M**).

### Parkvall (2008): Borrowability versus Genealogical Stability

Mikael Parkvall [58] proposes to distinguish between features which have a strong genealogical signal (‘a language needs to be “born with them” in order to have them’) from those that ‘may “come and go as they please’” (p.234). More precisely, he contrasts genealogically stable features defined as “a language either has it or lacks it, but whatever the case, contact or internal development is not going to change much” to unstable features, defined as “an easily borrowable or transferable characteristic, or for that matter, a feature easily gained or lost in contact” (p.235). Thus, it seems that his real focus is not on resistance to change whatever the cause of the change, be it internal (“mutation”-like processes) or external (language contact, selective pressures, random sampling or various types of constraints), but specifically on *resistance to borrowing*.

This is reinforced by the actual operationalisation of his definition (pp.235–238), which can be summarised as follows. First, he contrasts genealogical units (families and subfamilies from WALS [39]) to areal units (shown in his Map 1 on p.236). Second, for a given feature 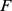 and a unit 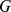 he computes the Herfindahl-Hirschman index (or Gini coefficient) defined as.
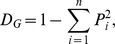
where 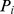 is the proportion of languages in 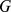 which have value 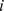 for the considered feature; in fact, this index is widespread in economics and is closely related to entropy (see Appendix A in [43]). The group’s homogeneity is obtained by taking the reciprocal of the Gini coefficient,



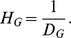



These homogeneities are then averaged over all the considered groups, resulting in the average homogeneity of feature 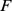 over families (when the groups are genealogical) 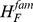 and areas (when the groups are areal) 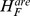. The actual measure of stability is the ratio of the two:
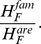



When using all the language families available in the WALS, the author obtains a stability estimate which we will denote here as 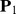, but he also considered only a subset composed of “only the most widely accepted families” (*Algonquian*, *Austronesian*, *Bantu*, *Dravidian*, *Indo-European*, *Iroquoian*, *Mayan*, *Mongolic*, *Semitic*, *Sino-Tibetan*, *Turkic* and *Uralic*; p. 240) resulting in an estimate denoted here as 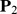. Those features scoring high on 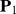 (and 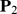) are those for which 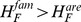, and thus those for which related languages tend to share the same value as opposed to those for which languages in contact share the same value.

Therefore, we propose that this method should be more appropriately seen as estimating *borrowability* (and its opposite, resistance to borrowing) and not as stability in the sense of resistance to change. The difference between them is readily seen if we think about a feature which is easy to borrow and yet not very stable.

### Wichmann & Holman (2009): Stable Features Tend to “Stay in the Family”

These authors [59] define the stability of a feature as “the probability that a given language remains unchanged with respect to the feature during [a fixed and arbitrary number of years; our note], that is, the feature undergoes neither internal change nor diffusion during the interval” (p.12), being thus explicitly an estimate of the feature’s resistance to change irrespective of the causes of change. They propose three slightly different methods for estimating the relative stabilities of WALS features. However, they conclude (by using computer simulations) that “metric C” performs the best, this is the only one we will describe and use here (denoted in the following as **W**).

The idea behind “metric C” is “that if one given feature more often tends to have the same value for languages that are related than does another given feature, then the first of the two may be considered to be more stable” (p.16) but the authors also correct for overall tendencies as well. For a feature 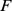 and a genealogical group 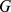 consisting of 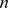 languages for which the feature is attested, they compute the proportion of pairs of languages sharing the same feature value 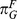. These proportions are then averaged across groups by weighting each group by 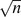 resulting in.
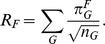



Likewise, they compute the proportion of pairs of unrelated languages sharing the same feature value 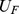 and their stability estimate 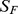 is obtained by correcting for this.
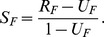



Thus, given that both 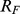 and 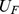 are bounded by 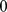 and 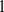, 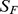 quantifies how much more similar related languages are than unrelated languages (on average), weighted by the maximum possible such difference. Therefore, 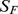 is a measure of genealogical stability irrespective of the actual causes of change.

### Comparing the Methods

For each of these methods we have extracted the estimated stabilities for each of the 142 structural features in WALS [39], as follows:

- **Cysouw, Albu & Dress (2008)**: for each of their three methods (**CM**, **CC** and **CR**) we have extracted the estimated stabilities from their paper’s Appendix D [43]. We use here the negative of **CR** to align it with the other methods;
- **Dediu (2011)**: we have extracted the agreed ranks (the scores on the first principal component) for the polymorphic features from Table S7 in the paper’s Electronic Supplementary Material [35]. We use here the negative of this estimate, **D**, to align it with the other methods;
- **Maslova (2002, 2004)**: we computed the stabilities, **M**, as described above and as detailed in the Supplementary Material Online;
- **Parkvall (2008)**: to allow comparability with the other methods, we have retained only the estimates for polymorphic features as computed using “all families” (denoted in the following by **P
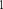
**) and using only the “most widely accepted families” (**P
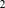
**), both extracted from his paper’s Appendix (pp.245–250) [58];
- **Wichmann & Holman (2009)**: we extracted the estimates produced by their “metric C” (denoted in the following by **W**) from their paper’s Appendix 1 (pp.43–46) [59].

These estimates are reported in Table 1 and each method’s coverage of the 142 WALS features is given in Table 2. **P
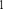
** and **M** cover the most features (136; 97.18%) while **D** covers only 68 (47.89%): these differences are explained by the minimal requirements of the methods and the threshold of maximally acceptable proportion of missing data used by the different authors. There are 62 (43.66%) shared features covered by all methods, namely (please note that WALS uses unique numeric identifiers for its features, given here in parantheses): *Consonant inventories* (1), *Vowel quality inventories* (2), *Voicing in plosives and fricatives* (4), *Uvular consonants* (6), *Glottalized consonants* (7), *Lateral consonants* (8), *Velar nasal*s (9), *Vowel nasalization* (10), *Front rounded vowels* (11), *Syllable structure* (12), *Tone* (13), *Absence of common consonants* (18), *Locus of marking in the clause* (23), *Locus of marking in possessive noun phrase*s (24), *Reduplication* (27), *Number of genders* (30), *Definite articles* (37), *Indefinite articles* (38), *Distance contrasts in demonstratives* (41), *Pronominal and adnominal demonstratives* (42), *Third-person pronouns and demonstratives* (43), *Gender distinctions in independent personal pronouns* (44), *Politeness distinctions in pronouns* (45), *Number of cases* (49), *Asymmetrical case marking* (50), *Ordinal numerals* (53), *Numeral classifiers* (55), *Position of pronominal possessive affixes* (57), *Obligatory possessive inflection* (58), *Possessive classification* (59), *Nominal and verbal conjunction* (64), *Perfective/imperfective aspect* (65), *Past tense* (66), *Future tense* (67), *Perfect* (68), *Morphological imperative* (70), *Optative* (73), *Overlap between situational and epistemic modal marking* (76), *Semantic distinctions of evidentiality* (77), *Suppletion according to tense and aspect* (79), *Verbal number and suppletio*n (80), *Order of subject and verb* (82), *Order of object and verb* (83), *Order of adposition and noun phrase* (85), *Order of genitive and noun* (86), *Order of adjective and noun* (87), *Order of numeral and noun* (89), *Order of degree word and adjective* (91), *Position of polar question particles* (92), *Position of interrogative phrases in content questions* (93), *Verbal person marking* (102), *Order of person markers on the verb* (104), *Passive constructions* (107), *Antipassive constructions* (108), *Applicative constructions* (109), *Symmetric and asymmetric standard negation* (113), *Predicative adjectives* (118), *Nominal and locational predication* (119), *Zero copula for predicate nominals* (120), *‘When’ clauses* (126), *‘Hand’ and ‘arm’* (129), *M-T pronouns* (136). *N-M pronouns* would also belong to this list if not for **P_2_**.

**Table 1:** Estimates of stability given by the various methods, converted to relative ranks (from 0.00 = most unstable, to 1.00 = most stable) for easiness of comparison between methods.

ID	Feature		Stability estimates (unstable to stable)							
	Name	Area	CM	CC	CR	D	P_1_	P_2_	W	M
1	Consonant inventories	P	0.21	0.41	0.11	0.01	0.36	0.56	0.10	0.02
2	Vowel quality inventories	P	0.43	0.79	0.75	0.50	0.65	0.38	0.48	0.40
3	Consonant-vowel ratio	P	–	–	–	0.04	0.48	0.29	0.18	0.07
4	Voicing in plosives and fricatives	P	0.75	0.55	0.44	0.22	0.22	0.49	0.24	0.15
5	Voicing and gaps in plosive systems	P	0.68	0.82	0.88	–	0.16	0.43	0.38	0.43
6	Uvular consonants	P	0.56	0.95	0.87	0.94	0.76	0.04	0.58	0.91
7	Glottalized consonants	P	0.66	0.93	0.91	0.75	0.57	0.85	0.67	0.70
8	Lateral consonants	P	0.55	0.85	0.58	0.46	0.39	0.80	0.46	0.49
9	Velar nasals	P	0.53	0.87	0.84	0.65	0.79	0.67	0.84	0.85
10	Vowel nasalization	P	0.29	0.95	0.74	0.97	0.74	0.96	0.91	0.88
11	Front rounded vowels	P	0.19	1.00	1.00	0.99	0.87	0.01	0.08	0.96
12	Syllable structure	P	0.31	0.76	0.60	0.37	0.16	0.10	0.28	0.43
13	Tone	P	0.46	0.92	0.90	0.90	0.29	0.56	0.75	0.69
14	Fixed stress locations	P	0.24	0.17	0.06	–	0.29	0.25	0.32	0.23
15	Weight-sensitive stress	P	0.26	0.32	0.16	–	0.48	0.35	0.13	0.12
16	Weight factors in weight-sensitive stress systems	P	0.14	0.20	0.02	–	0.50	0.29	0.07	0.03
17	Rhythm types	P	0.21	0.06	0.28	–	0.79	0.47	0.25	0.25
18	Absence of common consonants	P	0.61	0.99	0.99	1.00	0.92	0.96	0.88	0.99
19	Presence of uncommon consonants	P	0.41	0.97	0.92	–	0.60	0.21	0.16	0.68
20	Fusion of selected inflectional formatives	M	0.65	0.68	0.76	–	0.04	0.47	0.50	0.56
21	Exponence of selected inflectional formatives	M	0.87	0.39	0.54	–	0.34	0.44	0.87	0.46
22	Inflectional synthesis of the verb	M	0.38	0.15	0.10	–	0.39	0.35	0.09	0.01
23	Locus of marking in the clause	M	0.88	0.24	0.19	0.19	0.25	0.51	0.36	0.09
24	Locus of marking in possessive noun phrases	M	0.71	0.36	0.17	0.34	0.08	0.38	0.33	0.06
25	Locus of marking: whole-language typology	M	–	–	–	–	0.29	0.66	0.56	0.38
26	Prefixing versus suffixing in inflectional morphology	M	0.90	0.34	0.22	–	0.89	0.82	0.64	0.26
27	Reduplication	M	0.15	0.50	0.29	0.66	0.18	0.57	0.55	0.80
28	Case syncretism	M	0.78	0.77	0.59	–	0.50	0.62	0.96	0.97
29	Syncretism in verbal person/number marking	M	0.64	0.69	0.80	–	0.39	0.59	0.97	0.92
30	Number of genders	NC	0.84	0.64	0.18	0.56	0.67	0.92	0.99	0.86
31	Sex-based and non-sex-based gender systems	NC	0.88	0.74	0.50	–	0.89	0.99	1.00	1.00
32	Systems of gender assignment	NC	0.84	0.72	0.27	–	0.80	0.97	0.95	0.86
33	Coding of nominal plurality	NC	0.69	0.53	0.12	–	0.76	0.80	0.63	0.42
34	Occurrence of nominal plurality	NC	0.25	0.11	0.15	–	0.52	0.69	0.06	0.07
35	Plurality in independent personal pronouns	NC	0.51	0.46	0.05	–	0.22	0.43	0.47	0.13
36	Associative plural	NC	0.37	0.09	0.12	–	0.29	0.33	0.37	0.10
37	Definite articles	NC	0.29	0.29	0.09	0.10	0.52	0.31	0.13	0.04
38	Indefinite articles	NC	0.35	0.17	0.09	0.13	0.29	0.27	0.15	0.04
39	Inclusive/exclusive dist. in independent pronouns	NC	0.56	0.89	0.85	–	0.55	0.89	0.92	0.77
40	Inclusive/exclusive forms for ‘we’	NC	0.86	0.55	0.35	–	0.45	0.98	0.93	0.46
41	Distance contrasts in demonstratives	NC	0.02	0.40	0.34	0.32	0.34	0.71	0.12	0.32
42	Pronominal and adnominal demonstratives	NC	0.05	0.32	0.48	0.63	0.76	0.59	0.78	0.83
43	Third-person pronouns and demonstratives	NC	0.03	0.15	0.02	0.12	0.06	0.27	0.37	0.14
44	Gender dist. in independent personal pronouns	NC	0.64	0.91	0.74	0.49	0.80	0.88	0.76	0.74
45	Politeness distinctions in pronouns	NC	0.49	0.69	0.53	0.31	0.06	0.01	0.11	0.57
46	Indefinite pronouns	NC	0.58	0.22	0.29	–	0.67	0.06	0.52	0.64
47	Intensifiers and reflexive pronouns	NC	0.12	0.35	0.62	–	0.67	0.90	0.90	0.88
48	Person marking on adpositions	NC	0.68	0.81	0.73	0.35	–	–	0.61	0.62
49	Number of cases	NC	0.97	0.24	0.08	0.03	0.29	0.51	0.60	0.09
50	Asymmetrical case marking	NC	0.94	0.45	0.32	0.09	0.21	0.59	0.73	0.34
51	Position of case affixes	NC	0.95	0.53	0.56	–	0.57	0.82	0.71	0.60
52	Comitatives and instrumentals	NC	0.51	0.24	0.40	–	0.39	0.05	0.17	0.27
53	Ordinal numerals	NC	0.47	0.12	0.03	0.07	0.25	0.40	0.60	0.12
54	Distributive numerals	NC	0.16	0.02	0.20	–	0.63	0.41	0.69	0.59
55	Numeral classifiers	NC	0.04	0.43	0.57	0.84	0.73	0.23	0.59	0.87
56	Conjunctions and universal quantifiers	NC	0.05	0.04	0.31	–	0.18	0.12	0.35	0.18
57	Position of pronominal possessive affixes	NC	0.62	0.39	0.24	0.54	0.98	0.87	0.86	0.62
58	Obligatory possessive inflection	NS	0.43	0.89	0.88	0.93	0.29	0.89	0.01	0.72
59	Possessive classification	NS	0.59	0.65	0.40	0.76	0.73	0.86	0.02	0.08
60	Genitives, adjectives, and relative clauses	NS	0.22	0.05	0.60	–	0.39	0.34	0.07	0.05
61	Adjectives without nouns	NS	0.09	0.02	0.77	–	0.45	0.27	0.82	0.22
62	Action nominal constructions	NS	0.08	0.07	0.01	–	0.63	0.21	0.65	0.17
63	Noun phrase conjunction	NS	0.59	0.19	0.89	–	0.03	0.68	0.83	0.76
64	Nominal and verbal conjunction	NS	0.28	0.30	0.45	0.43	0.18	0.19	0.21	0.45
65	Perfective/imperfective aspect	VC	0.41	0.57	0.66	0.68	0.11	0.35	0.54	0.70
66	Past tense	VC	0.83	0.35	0.39	0.47	0.86	0.72	0.79	0.58
67	Future tense	VC	0.74	0.57	0.71	0.59	0.48	0.18	0.34	0.59
68	Perfect	VC	0.72	0.47	0.65	0.18	0.04	0.14	0.25	0.22
69	Position of tense-aspect affixes	VC	0.92	0.66	0.57	–	0.95	0.75	0.72	0.61
70	Morphological imperative	VC	0.76	0.67	0.50	0.21	0.71	0.68	0.32	0.37
71	Prohibitive	VC	0.18	0.43	0.07	–	0.65	0.31	0.28	0.20
72	Imperative-hortative systems	VC	0.24	0.75	0.61	–	0.52	0.15	0.17	0.44
73	Optative	VC	0.11	0.98	0.95	0.96	0.81	0.14	0.90	0.96
74	Situational possibility	VC	0.10	0.67	0.52	–	0.44	0.24	0.43	0.53
75	Epistemic possibility	VC	0.55	0.53	0.26	–	0.06	0.15	0.41	0.24
76	Overlap b/w situational & epistemic modal marking	VC	0.33	0.38	0.33	0.28	0.25	0.03	0.04	0.11
77	Semantic distinctions of evidentiality	VC	0.91	0.74	0.86	0.40	0.76	0.25	0.42	0.36
78	Coding of evidentiality	VC	0.91	0.61	0.70	–	0.60	0.22	0.22	0.31
79	Suppletion according to tense and aspect	VC	0.38	0.72	0.36	0.62	0.11	0.64	0.79	0.66
80	Verbal number and suppletion	VC	0.26	0.93	0.82	0.53	0.97	1.00	0.62	0.63
81	Order of subject, object, and verb	WO	0.98	0.41	0.46	–	0.90	0.74	0.81	0.50
82	Order of subject and verb	WO	0.85	0.96	0.96	0.79	0.95	0.74	0.53	0.90
83	Order of object and verb	WO	1.00	0.84	0.98	0.74	0.99	0.56	0.94	0.84
84	Order of object, oblique, and verb	WO	0.81	0.10	0.63	–	0.48	0.51	0.87	0.57
85	Order of adposition and noun phrase	WO	0.99	0.84	0.98	0.57	0.96	0.78	0.97	0.93
86	Order of genitive and noun	WO	0.98	0.88	0.93	0.87	0.99	0.88	0.93	0.91
87	Order of adjective and noun	WO	0.79	0.83	0.83	0.51	0.87	0.76	0.76	0.83
88	Order of demonstrative and noun	WO	0.96	0.90	0.81	–	0.92	0.77	0.66	0.71
89	Order of numeral and noun	WO	0.79	0.79	0.68	0.71	0.82	0.91	0.85	0.89
90	Order of relative clause and noun	WO	0.57	0.50	0.53	–	0.22	0.41	0.84	0.78
91	Order of degree word and adjective	WO	0.43	0.15	0.64	0.60	0.63	0.64	0.49	0.52
92	Position of polar question particles	WO	0.17	0.26	0.13	0.06	0.67	0.33	0.29	0.21
93	Position of interrogative phrases in content questions	WO	0.53	0.73	0.67	0.44	0.76	0.78	0.66	0.81
94	Order of adverbial subordinator and clause	WO	0.89	0.32	0.43	–	0.18	0.49	0.69	0.54
95	Relationship between OV/VO and PREP/POST	WO	–	–	–	0.38	0.91	0.59	–	0.75
96	Relationship between OV/VO and N REL/REL N	WO	–	–	–	0.25	0.34	0.38	–	0.67
97	Relationship between OV/VO and ADJ-N/N-ADJ	WO	–	–	–	0.15	0.70	0.65	–	0.54
98	Alignment of case marking of full noun phrases	SC	0.95	0.61	0.36	–	0.84	0.69	0.71	0.39
99	Alignment of case marking of pronouns	SC	0.83	0.48	0.37	–	0.44	0.76	0.78	0.29
100	Alignment of verbal person marking	SC	0.74	0.63	0.33	–	0.57	0.83	0.51	0.41
101	Expression of pronominal subjects	SC	0.78	0.50	0.14	–	0.69	0.54	0.43	0.41
102	Verbal person marking	SC	0.93	0.71	0.81	0.24	0.21	0.19	0.19	0.28
103	Third-person zero of verbal person marking	SC	0.81	0.61	0.55	–	0.36	0.49	0.31	0.20
104	Order of person markers on the verb	SC	0.70	0.61	0.47	0.16	0.71	0.46	0.57	0.38
105	Ditransitive constructions: the verb ‘give’	SC	0.31	0.32	0.16	–	0.93	0.85	0.19	0.14
106	Reciprocal constructions	SC	0.48	0.15	0.25	–	0.11	0.56	0.22	0.30
107	Passive constructions	SC	0.20	0.97	0.97	0.69	0.82	0.81	0.39	0.82
108	Antipassive constructions	SC	0.37	0.86	0.49	0.82	0.13	0.65	0.23	0.47
109	Applicative constructions	SC	0.40	0.50	0.23	0.26	0.16	0.71	0.54	0.30
110	Periphrastic causative constructions	SC	0.13	0.09	0.64	–	0.11	0.44	0.10	0.33
111	Nonperiphrastic causative constructions	SC	0.20	0.91	0.47	–	0.29	0.84	0.74	0.80
112	Negative morphemes	SC	0.77	0.54	0.22	–	0.84	0.41	0.34	0.36
113	Symmetric and asymmetric standard negation	SC	0.60	0.78	0.78	0.29	0.39	0.61	0.27	0.17
114	Subtypes of asymmetric standard negation	SC	0.46	0.45	0.05	–	0.39	0.53	0.45	0.16
115	Negative indefinite pronouns and predicate negation	SC	0.40	0.28	0.94	–	0.08	0.11	0.03	0.73
116	Polar questions	SC	0.73	0.65	0.43	–	0.48	0.23	0.29	0.51
117	Predicative possession	SC	0.17	0.04	0.04	–	0.71	0.62	0.51	0.28
118	Predicative adjectives	SC	0.66	0.29	0.30	0.72	0.96	0.94	0.99	0.95
119	Nominal and locational predication	SC	0.63	0.59	0.79	0.91	0.91	0.94	0.98	0.99
120	Zero copula for predicate nominals	SC	0.30	0.57	0.71	0.85	0.88	0.10	0.40	0.79
121	Comparative constructions	SC	0.52	0.01	0.67	–	0.02	0.15	0.89	0.72
122	Relativization on subjects	CS	0.33	0.38	0.72	–	0.57	0.11	0.68	0.93
123	Relativization on obliques	CS	0.09	0.07	0.42	–	0.29	0.07	0.57	0.35
124	want complement clauses	CS	0.13	0.13	0.21	–	0.57	0.73	0.40	0.33
125	Purpose clauses	CS	0.35	0.21	0.41	–	0.16	0.49	0.75	0.49
126	when clauses	CS	0.70	0.24	0.26	0.41	0.63	0.37	0.46	0.48
127	Reason clauses	CS	0.45	0.28	0.38	–	0.13	0.10	0.70	0.64
128	Utterance complement clauses	CS	0.07	0.26	0.51	–	0.55	0.17	0.04	0.55
129	‘hand’ and ‘arm’	L	0.02	0.12	0.69	0.78	0.43	0.04	0.63	0.65
130	‘finger’ and ‘hand’	L	0.01	0.20	0.95	–	0.34	0.93	0.49	0.94
131	Numeral bases	L	0.49	0.47	0.19	–	0.54	0.93	0.26	0.25
132	Number of nonderived basic colour categories	L	–	–	–	–	0.06	0.29	0.14	0.19
133	Number of basic colour categories	L	–	–	–	–	0.11	0.18	0.05	0.01
134	‘green’ and ‘blue’	L	–	–	–	–	0.39	0.08	0.44	0.51
135	‘red’ and ‘yellow’	L	–	–	–	–	0.60	–	0.01	0.78
136	M-T pronouns	L	0.27	0.77	0.91	0.81	1.00	0.99	0.20	0.98
137	N-M pronouns	L	0.06	0.81	0.77	0.88	0.84	–	0.81	0.75
138	Etymology of ‘tea’	L	0.33	0.02	0.84	–	0.01	0.07	–	0.67
139	Irregular Negatives in Sign Languages	SL	–	–	–	–	–	–	–	–
140	Question Particles in Sign Languages	SL	–	–	–	–	–	–	–	–
141	Writing Systems	O	–	–	–	–	–	–	–	–
142	Paralinguistic usages of clicks	O	–	–	–	–	0.01	0.02	–	–

**ID** and **Name** are as in WALS [39]. **D** is [35]’s 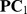, 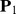 and 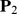 are [58]’s “all families” and “accepted families only”, **W** is [59]’s “metric C”, **CM**, **CC** and **CR** are [43]’s “Mantel”, “Coherence” and “Rank” methods, **M** represents estimates of Maslova’s stability (as implemented by us). We used **-D** and **-CR** to ensure that all estimates have the same directionality. The WALS **Area** is given as **P**honology, **M**orphology, **N**ominal **C**ategories, **N**ominal **S**yntax, **V**erbal **C**ategories, **W**ord **O**rder, **S**imple **C**lauses, **C**omplex **S**entences, **L**exicon, **S**ign **L**anguages and **O**ther. See text for details.

**Table 2:** Coverage of the WALS features.

Coverage	Method							
	CM	CC	CR	D	P_1_	P_2_	W	M
Number Percent	129	129	129	68	138	136	134	138
	90.85%	90.85%	90.85%	47.89%	97.18%	95.77%	94.37%	97.18%
	Shared among all methods							
Number Percent	62							
	43.66%							

Shown are each method’s coverage – i.e., the method provides an estimated stability – as number and percent of the 142 WALS features.

Conceptually, these methods propose quite different approaches to the structural stability of language. Dediu (2011) [35] uses a standard concept from evolutionary biology, in which stability is equated with resistance to change (irrespective of the causes of change) while languages evolve following an assumed tree-like history. Stable features are those with a low rate of change. A related idea, but much simpler and ignoring many possible problematic issues, is proposed by Wichmann & Holman (2009) [59], which take stable features to be those that tend to share values within families rather than across them. Maslova’s method [52]–[55] also shares fundamental insights with Dediu (2011) and Wichmann & Holman (2009) in the sense that stability is understood in a genealogical context. Parkvall (2008) [58] estimates something which would probably be better called non-borrowability rather than stability in the sense that features high on this scale are those shared more within genealogical units than within linguistic areas. Finally, Cysouw, Albu & Dress (2008) [43] describe a method which apparently has no genealogical component, whereby they estimate the consistence of a feature with the overall pattern given by many features.

## Results

All analyses and graphs were realised using R [57].

### Pairwise Relationships between Methods

**Figure 1:**
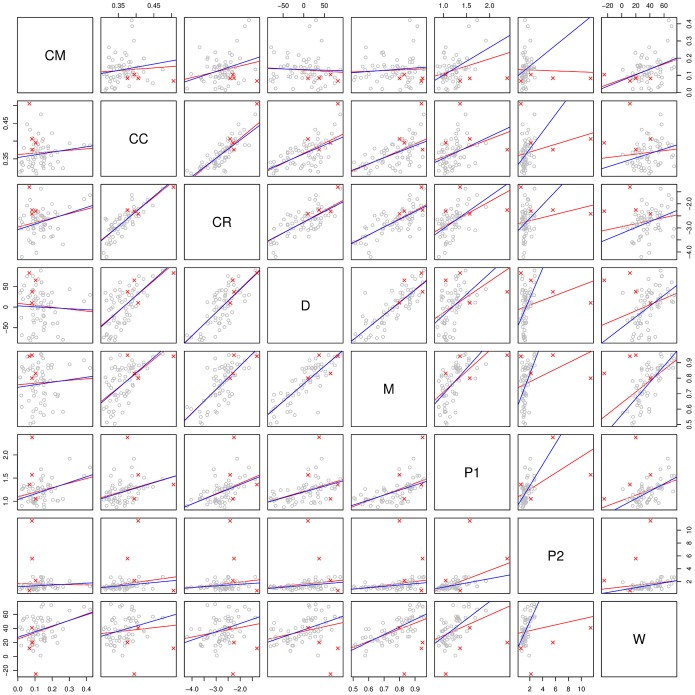
Relationship between different stability estimates. Each panel shows the scatterplot of the stability estimates for the shared features produced by a pair of methods (grey dots) and the identified outliers (red crosses; see text for details). The regression lines with the outliers (red) and without (blue) have been drawn for convenience.

Using all 62 features shared across all methods, the relationships between all pairs of methods are represented in the scatterplots in Figure 1. Table 3 shows the pairwise correlations (Pearson’s 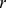 and Spearman’s 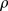) between the stability estimates. For each pair of methods, we inspected the scatterplots and regression diganostic plots (using R’s lm() function; Residuals vs Fitted, QQ-plot and Leverage) in order to identify outliers. Given the small number of shared features across all methods, we applied a conservative approach by selecting only those features that were strong outliers for several pairs of methods, identifying the following features: *Verbal Number and Suppletion* (80), *Obligatory Possessive Inflection* (58), *M-T Pronouns* (136), and *Front Rounded Vowels* (11); see also Figure 1. Without these outliers the correlations do not change much (see Table 4), except for 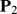, as it takes an extreme position for almost all outlier features.

**Table 3:** Pairwise correlations between stability estimates for all shared features.

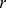 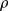 /	CM	CC	CR	D	M	P1	P2	W
CM	–	0.08	0.25*	**−0.08**	**0.06**	0.29*	−0.03	0.36**
		0.534	**0.047**	0.523	0.636	**0.021**	0.838	**0.003**
CC	0.10	–	0.82**	0.65**	0.59**	0.33**	0.21	0.12
	0.428		**<2.2⋅10^−16^**	**8.34⋅10^−9^**	**2.95⋅10^−7^**	**0.008**	0.108	0.353
CR	0.12	0.83**	–	0.73**	0.72**	0.49**	0.17	0.21
	0.346	**<22⋅10^−16^**		**7.78⋅10^−12^**	**2.48⋅10^−11^**	**5.34⋅10^−5^**	0.189	0.102
D	−0.177	0.60**	0.68**	–	0.83**	0.45**	0.20	0.31*
	0.177	**1.35⋅10^−7^**	**<2.2⋅10^−16^**		**<2.2⋅10^−16^**	**2.20⋅10^−16^**	0.127	**0.014**
M	−0.01	0.59**	0.68**	0.82**	–	0.68**	0.39**	0.62**
	0.941	**2.41⋅10^−7^**	**<2.2⋅10.41⋅10^−16^**	**<2.2⋅10^−16^**		**1.52⋅10^−9^**	**0.002**	**4.70⋅10^−8^**
P1	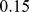 0.15	0.42**	0.54**	0.52**	0.58**	–	0.49**	0.41**
	0.254	**7.02⋅10^−4^**	**4.20⋅10^−6^**	**1.32⋅10^−5^**	**5.37⋅10^−7^**		**4.89⋅10^−5^**	**8.63⋅10^−4^**
P2	0.23	0.33**	0.18	0.29*	0.25	0.48**	–	0.16
	0.075	**0.008**	0.154	**0.020**	0.053	**7.25⋅10^−5^**		0.215
W	0.28*	0.23	0.25*	0.39**	0.56**	0.51**	0.46**	–
	0.028	0.072	**0.048**	**0.001**	**1.876⋅10^−6^**	**1.83⋅10^−5^**	**1.87⋅10^−4^**	

Upper diagonal: Pearson’s 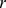; lower diagonal: Spearman’s 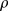; within cells, upper line is the correlation estimate (* stands for significant correlation at 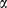-level = 0.05, **the correlation is significant at 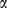-level = 0.01; all significant correlations are in **bold**) and the lower line is the 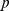-value.

**Table 4:** Pairwise correlations between stability estimates excluding the outliers.

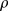 /*r*	CM	CC	CR	D	M	P1	P2	W
CM	–	0.17	0.33**	−0.04	0.11	0.45**	0.20	0.37**
		0.200	**0.011**	0.754	0.420	**3.96⋅10^−4^**	0.123	**0.004**
CC	0.19	–	0.80**	0.62**	0.58**	0.38**	0.36**	0.30*
	0.150		**2.20⋅10^−1^**	**1.16⋅10^−7^**	**1.12⋅10^−6^**	**0.003**	**0.006**	**0.018**
CR	0.21	0.81**	–	0.71**	0.70**	0.53**	0.27*	0.38**
	0.111	**3.01⋅10^−15^**		**3.07⋅10^−10^**	**4.21⋅10^−10^**	**1.48⋅10^−5^**	**0.043**	**0.003**
D	−0.12	0.57**	0.65**	–	0.83**	0.51**	0.44**	0.51**
	0.356	**1.71⋅10^−6^**	**3.76⋅10^−8^**		**4.44⋅10^−16^**	**4.23⋅10^−5^**	**5.75⋅10^−4^**	**2.68⋅10^−5^**
M	0.06	0.58**	0.66**	0.82**	–	0.60**	0.48**	0.75**
	0.625	**1.30⋅10^−6^**	**3.07⋅10^−8^**	**<2.2⋅10^−16^**		**5.22⋅10^−7^**	**1.59⋅10^−4^**	**6.71⋅10^−12^**
P1	0.24	0.39**	0.52**	0.53**	0.67**	–	0.55**	0.63**
	0.061	**0.002**	**2.43⋅10^−5^**	**1.38⋅10^−5^**	**8.62⋅10^−9^**		**7.97⋅10^−6^**	**6.87⋅10^−8^**
P2	0.29*	0.35**	0.17	0.32*	0.42**	0.50**	–	0.61**
	**0.026**	**0.007**	0.212	**0.014**	**0.001**	**5.35⋅10^−5^**		**3.74⋅10^−7^**
W	0.25	0.33**	0.37**	0.55**	0.77**	0.62**	0.54**	–
	0.051	**0.010**	**0.003**	**6.27⋅10^−6^**	**3.76⋅10^−13^**	**2.04⋅10^−7^**	**1.01⋅10^−5^**	

Upper diagonal: Pearson’s 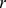; lower diagonal: Spearman’s 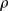; within cells, upper line is the correlation estimate (*stands for significant correlation at 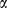-level = 0.05, **the correlation is significant at 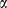-level = 0.01; all significant correlations are in **bold**) and the lower line is the 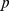-value.

**Figure 2:**
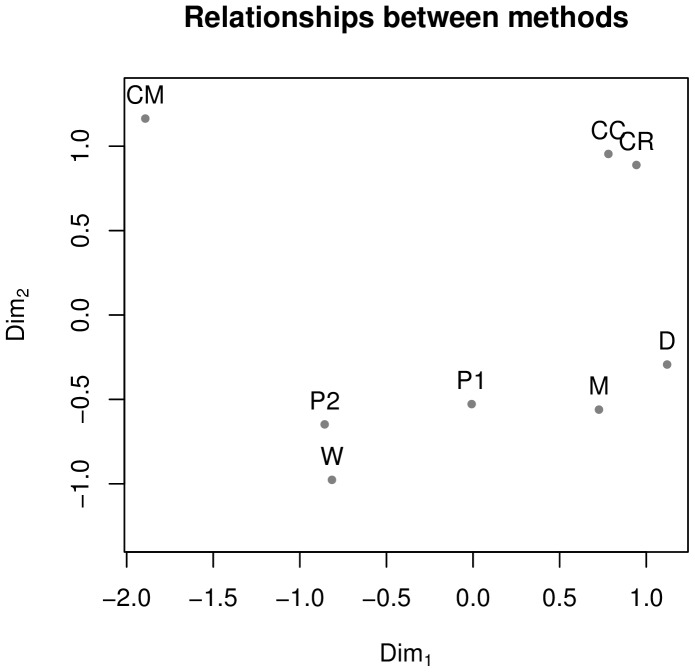
Relationships between methods. The distances between methods computed in the 62-dimensional space defined by the relative ranks of all shared features projected using classic Multidimensional Scaling (MDS). The results excluding the outlier features are extremely similar.

Figure 2 shows a different view of the relationship between methods: in the 62-dimensional space determined by the shared features, each method represents a single point with coordinates given by the relative ranks of the features as computed by the method. In order to meaningfully compare the feature stabilities across methods, we converted them to relative ranks between 0.0 (most unstable feature) and 1.0 (most stable feature). We did this for each method separately, by first computing the ranks of the method’s actual stability estimates for all the features that the method provided estimates for, and then by normalizing these ranks between 0 and 1 using the formula 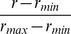 where 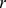 is a given feature’s rank and 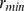 and 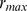 are the smallest and largest ranks respectively. In this space we computed all pairwise Euclidean distances between the methods and projected these distances on two dimensions using classical multidimensional scaling (MDS) [60], resulting in Figure 2. A small distance between two methods means that they tend to estimate the same relative stabilities for all features, while a large distance signals disagreements between methods. The maximum possible distance in this space is 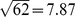 but the distances between methods are between 1.34 and 3.43 with a mean of 2.43, suggesting again that the methods agree better than expected by chance. This was confirmed by randomly generating 10,000 sets of seven points in this 62-dimensional space and comparing the distribution of these generated distances to the observed distances between methods: both min and mean observed distances are much smaller than expected (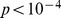), while the maximum distance is also smaller but within the distribution of maximum random distances (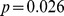). It can be seen that **CC** and **CR** form a tight cluster, as do **P_2_** and **W**. Further, **D** and **M** are relatively close together, while **CM** is a clear outlier.

### Principal Components Analysis

To better understand the relationships between the stability estimates produced by these methods we have conducted a Principal Components Analysis [61] both on the full set of shared features and on the set excluding the outliers, using the actual stability estimates provided by each method.

On the full set of 62 shared features, the first four Principal Components explain in total 89.2% of the variation (Table 5). 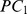 explains 48.3% and represents the agreement between all methods (their loadings have the same sign). 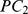 explains 17.2% and contrasts **CC**, **CR** and **D** on one hand, and **CM**, **P_1_**, **P_2_** and **W** on the other (excluding **M**, which has a loading close to zero on this component). 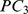 explains 13.5% and makes a further distinction between the three methods in [43] (**CM**, **CC** and **CR**) and the two methods in [58] (**P_1_** and **P_2_**). Finally, 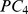 explains 10.2% and contrasts **D**, **W** and **M** with the other methods.

**Table 5:** Principal components analysis on the full set of 62 shared features.

Loadings	*PC*_1_	*PC*_2_	*PC*_3_	*PC*_4_
*% variance explained*	*48.3%*	*17.2%*	*13.5%*	*10.2%*
**CM**	**0.13**	**0.61**	**0.44**	**0.46**
**CC**	**0.39**	**−** ***0.33***	**0.16**	**0.32**
**CR**	**0.44**	**−** ***0.19***	**0.24**	**0.29**
**D**	**0.43**	**−** ***0.28***	0.04	−*0.25*
**M**	**0.46**	−0.04	0.02	−*0.36*
**P_1_**	**0.36**	**0.31**	−*0.31*	**0.15**
**P_2_**	**0.20**	**0.19**	−*0.79*	**0.28**
**W**	**0.26**	**0.53**	0.08	−*0.55*

Significant loadings with the same sign significant are in **bold** and *italic* to increase clarity.

The first principal component, explaining by far most of the variance (48.3%), represents the agreement between all these highly different methods. The following components also make interesting distinctions, such as the grouping together of the strongly phylogenetic method **D** with two of the strongly non-genealogical methods **CC** and **CR** (component 2), the identification of [58]’s special concept of “borrowability” (**P_1_n** and **P_2_**) versus [43]’s “consistency” (**CM**, **CC** and **CR**) (component 3), and the recognition that **D**, **W** and **M** methods are fundamentally similar, even if differing widely in details (component 4).

When excluding the outliers, the first four principal components explain 90.5% of the variation (Table 6). The first principal component, 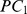, explains 55.8% of the variation and represents the agreement between all methods with similar loadings to the previous case. 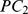 explains 16.1% and distinguishes **CC**, **CR**, **D** and **M** on one hand, from the **CM**, **P_1_**, **P_1_** and **W**, on the other, in a pattern similar to the previous case. However, 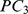 (12.2% of the variance) and 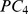 (6.8%) differ from the ones found using all shared features; 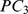 still distinguishes [43]’s “consistency” (but not [58]’s “borrowability”).

**Table 6:** Principal components analysis excluding the outliers.

Loadings	*PC*_1_	*PC*_2_	*PC*_3_	*PC*_4_
*% variance explained*	*55.8%*	*16.1%*	*12.2%*	*6.8%*
**CM**	**0.18**	−*0.60*	−*0.60*	0.03
**CC**	**0.35**	**0.36**	−*0.33*	−*0.46*
**CR**	**0.39**	**0.27**	−*0.43*	0.03
**D**	**0.39**	**0.36**	**0.17**	**0.17**
**M**	**0.42**	**0.16**	**0.20**	**0.37**
**P_1_**	**0.37**	−*0.32*	−0.02	0.08
**P_2_**	**0.31**	−*0.27*	**0.43**	−*0.72*
**W**	**0.37**	−*0.34*	**0.30**	**0.31**

Significant loadings with the same sign significant are in **bold** and *italic* to increase clarity.

**Figure 3:**
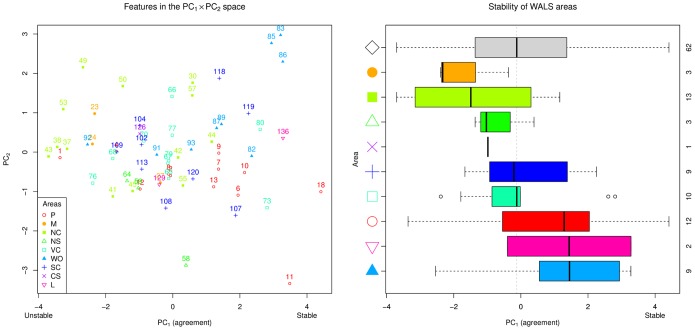
Distribution of features’ stabilities across methods. Left panel: a ll shared features (given by their numeric WALS unique ID; see Table 1) plotted on the 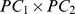. Right panel: the distribution of the stability across the WALS areas, with the number of features of each type shown on the right. 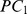 represents the strong inter-method agreement and varies from the unstable (left) to stable (right); the actual scales of the axes are arbitrary. The colours and symbols represent the WALS areas (see Table 1 for details), with the open diamond 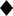 representing all shared features together. The results excluding the outlier methods are extremely similar.

Figure 3 (left panel) shows all shared features in the 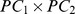 space, capturing together 65.5% of the variation between methods. Features that cluster together are features that show similar stability estimates across methods. The right panel compares the stability of the various WALS areas and shows that Word Order features tend to be the most stable, with Phonology covering the whole spectrum. An one-way ANOVA shows that the areas differ in their average stability (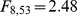, 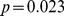), but a post-hoc pairwise comparison using Tukey’s HSD shows that only Word Order – Nominal Categories survive the multiple testing correction (adjusted 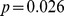), most probably due to the small numbers of features included.

### The Agreement and Differences between Methods

The pairwise correlations and the Principal Components Analysis strongly suggest that there is an important agreement between these different methods in what concerns the stability of the shared features.

Table 7 shows the shared features sorted from the most stable to the most unstable by their scores on the first principal component when using all shared features, 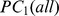, and then by the first principal component when excluding the outlier features, 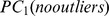. The correlation between 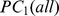 and 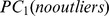 is 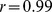, 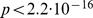.

**Table 7:** The shared features sorted by their agreed stabilities from the most stable to the most unstable.

Rank	ID	Name	Abbr. name	PC_1_	PC_1_*	PC_2_	PC_3_	PC_4_
1	18	Absence of Common Consonants	AbsComC	4.41	5.16	−1.01	0.31	0.32
2	11	Front Rounded Vowels	FrRoundV	3.48	NA	−3.34	1.16	1.09
3	136	M-T Pronouns	MTPron	3.28	NA	0.35	−3.51	1.15
4	86	Order of Genitive and Noun	GenN	3.28	4.17	2.30	0.46	0.57
5	83	Order of Object and Verb	OV	3.21	3.75	2.97	1.97	1.33
6	85	Order of Adposition and Noun Phrase	AdposNP	2.94	3.63	2.77	1.69	0.84
7	73	The Optative	Optative	2.81	2.70	−1.41	0.63	−1.09
8	80	Verbal Number and Suppletion	VnumSupp	2.61	NA	0.58	−5.61	1.94
9	82	Order of Subject and Verb	SV	2.35	2.59	−0.10	0.66	0.83
10	119	Nominal and Locational Predication	NomLocPred	2.25	3.21	0.98	−0.51	−1.49
11	10	Vowel Nasalization	VowelN	2.14	2.94	−0.52	−0.49	−0.94
12	6	Uvular Consonants	UvulC	1.94	1.74	−1.09	0.77	−0.37
13	107	Passive Constructions	PassiveC	1.87	2.01	−1.61	0.10	0.50
14	89	Order of Numeral and Noun	NumN	1.45	2.12	0.71	0.04	−0.44
15	118	Predicative Adjectives	PredAdj	1.38	2.47	1.87	−0.93	−1.73
16	9	The Velar Nasal	VelarN	1.37	1.58	−0.03	0.35	−0.65
17	7	Glottalized Consonants	GlotC	1.36	1.70	−0.43	0.45	0.16
18	87	Order of Adjective and Noun	AdjN	1.31	1.70	0.61	0.38	−0.06
19	13	Tone	Tone	1.22	1.24	−0.88	0.69	−0.60
20	44	Gender Dist. in Indep. Personal Pronouns	GenDIPersP	1.16	1.74	0.27	0.01	−0.04
21	120	Zero Copula for Predicate Nominals	ZeroCopPredNom	0.60	0.41	−0.68	0.01	−0.61
22	30	Number of Genders	NoGen	0.59	1.46	1.76	−0.08	−1.30
23	57	Position of Pronominal Possessive Affixes	PosProPAff	0.58	1.31	1.44	−0.90	−0.76
24	93	Pos. of Inter. Phrases in Content Questions	IntPhCQ	0.55	0.86	0.07	−0.10	−0.39
25	58	Obligatory Possessive Inflection	OlbPosInfl	0.39	NA	−2.87	−0.16	1.52
26	55	Numeral Classifiers	NumClas	0.30	0.18	−0.85	−0.37	−1.58
27	42	Pronominal and Adnominal Demonstratives	PadDem	0.16	0.30	−0.14	−0.56	−1.83
28	77	Semantic Distinctions of Evidentiality	SemDistEv	−0.02	−0.02	0.43	0.90	1.19
29	66	The Past Tense	PastTense	−0.02	0.39	1.41	0.09	−0.52
30	2	Vowel Quality Inventories	Vowel	−0.07	−0.11	−0.60	0.27	0.21
31	79	Suppletion According to Tense and Aspect	SuppTAsp	−0.12	0.01	−0.22	0.23	−1.24
32	65	Perfective/Imperfective Aspect	PerfImpAsp	−0.12	−0.25	−0.67	0.44	−0.77
33	8	Lateral Consonants	LatC	−0.12	0.09	−0.56	0.09	0.22
34	67	The Future Tense	FutTense	−0.15	−0.31	−0.28	0.61	0.06
35	108	Antipassive Constructions	AntipassiveC	−0.21	−0.30	−1.42	0.23	−0.04
36	27	Reduplication	Redup	−0.37	−0.41	−0.78	−0.12	−1.32
37	129	Hand and Arm	HandArm	−0.40	−0.64	−0.83	−0.17	−1.77
38	91	Order of Degree Word and Adjective	DegWAdj	−0.48	−0.42	−0.07	−0.13	−0.61
39	70	The Morphological Imperative	MorphImp	−0.86	−0.70	0.28	0.13	0.80
40	113	Symm. and Asymmetric Standard Negation	SymAsymStNeg	−0.92	−0.91	−0.43	0.41	1.01
41	102	Verbal Person Marking	VpersM	−0.93	−1.08	0.19	1.20	1.48
42	12	Syllable Structure	SylStr	−0.98	−1.23	−0.94	0.41	0.14
43	126	When’ Clauses	WhenC	−0.98	−0.96	0.47	0.08	−0.29
44	104	Order of Person Markers on the Verb	PersMV	−0.99	−0.84	0.67	0.18	0.39
45	59	Possessive Classification	PosClas	−1.03	−0.84	−0.91	−0.46	1.31
46	45	Politeness Distinctions in Pronouns	PolitDPron	−1.20	−1.62	−0.99	0.64	0.22
47	64	Nominal and Verbal Conjunction	NomVConj	−1.36	−1.61	−0.73	0.08	−0.35
48	50	Asymmetrical Case−Marking	AsymCaseM	−1.49	−1.20	1.68	0.77	0.38
49	109	Applicative Constructions	ApplicativeC	−1.67	−1.50	0.01	−0.17	−0.32
50	4	Voicing in Plosives and Fricatives	VoicPF	−1.69	−1.69	0.02	0.37	0.84
51	41	Distance Contrasts in Demonstratives	DistCDem	−1.79	−1.81	−1.12	−0.72	−0.17
52	68	The Perfect	Perfect	−1.79	−1.99	−0.16	0.75	0.65
53	23	Locus of Marking in the Clause	LmarkC	−2.33	−2.20	0.98	0.33	0.63
54	76	Overlap b/w Sit. and Epistemic Modal Mark.	OvSitEpi	−2.39	−2.72	−0.79	0.03	0.65
55	24	Locus of Marking in Possessive Noun Phrases	LmarkPNP	−2.40	−2.40	0.21	0.24	0.31
56	92	Position of Polar Question Particles	PolQPart	−2.54	−2.53	0.19	−0.67	−0.12
57	49	Number of Cases	Ncases	−2.68	−2.37	2.15	0.52	0.71
58	37	Definite Articles	DefArt	−3.15	−3.18	0.08	−0.56	0.46
59	53	Ordinal Numerals	OrdNum	−3.26	−3.08	1.09	−0.50	−0.62
60	1	Consonant Inventories	Cons	−3.36	−3.32	−0.14	−0.62	0.72
61	38	Indefinite Articles	IndefArt	−3.44	−3.49	0.13	−0.46	0.38
62	43	Third Person Pronouns and Demonstratives	P3PrDem	−3.70	−3.75	−0.11	−0.85	−0.99

The **Rank** represents the feature’s rank from the most “stable” (top) to the most “unstable” (bottom), the **ID** is the feature’s numeric identifier in WALS [39], **Name** is the feature’s full name while **Abbr. name** is the abbreviated name, and 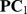 and 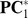 the feature’s score on the first principal component representing the agreement between all methods and excluding outliers, respectively; 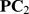 – 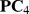 are the loadings on principal components 2, 3 and 4 using all shared features. See text for details.

**Figure 4:**
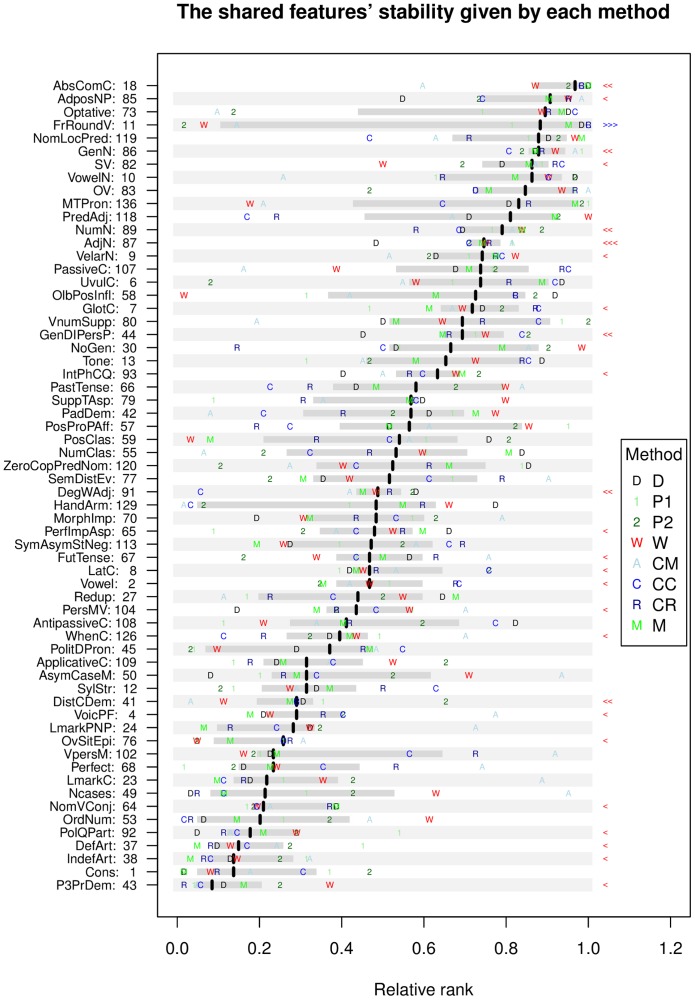
The features’ stabilities. The stabilities (as relative ranks from 0.0 = most unstable to 1.0 = most stable) of the shared features as estimated by all methods. Shown are the median stability (black thick lines), the interquartile range (IQR; light gray) and the individual method estimates (D, 1, 2, W, A, C, R, and M – see legend for details). The features with a significantly smaller or larger IQR than expected by chance are marked with red “<” and blue “>” symbols respectively on the right-hand side of the figure, with the number of symbols being one for 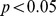, two for 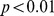 and three for 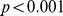; please note that this is before the multiple testing correction, after which only the features with 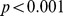, 11 and 87, survive (see text for details). The features are represented by transparently abbreviated names derived from their full WALS names (see Table 8) and their WALS unique IDs.

Figure 4 shows the shared features ordered by their median relative rank across all methods, an indication of the agreement between methods for each feature (the interquartile range, IQR), as well as the actual estimate given by each individual method. Table 8 shows the features ordered by the disagreement between methods (IQR). It can be seen that despite a clear overall concordance between methods (as shown by the first principal component), the agreement is far from perfect, and there are clear differences between methods both overall (reflected in the second, third and fourth Principal Components, and in the patterns of inter-method correlations and distances), and in what concerns the estimated stability of individual features.

**Table 8:** Differences in stability among methods for individual features.

ID	Name	Abbr. name	IQR	Range
87	Order of Adjective and Noun	AdjN	0.08	0.33
86	Order of Genitive and Noun	GenN	0.09	0.18
91	Order of Degree Word and Adjective	DegWAdj	0.11	0.52
18	Absence of Common Consonants	AbsComC	0.12	0.40
41	Distance Contrasts in Demonstratives	DistCDem	0.14	0.62
44	Gender Distinctions in Independent Personal Pronouns	GenDIPersP	0.15	0.40
89	Order of Numeral and Noun	NumN	0.15	0.31
93	Position of Interrogative Phrases in Content Questions	IntPhCQ	0.15	0.33
9	The Velar Nasal	VelarN	0.16	0.31
43	Third Person Pronouns and Demonstratives	P3PrDem	0.16	0.35
82	Order of Subject and Verb	SV	0.16	0.44
37	Definite Articles	DefArt	0.17	0.40
92	Position of Polar Question Particles	PolQPart	0.17	0.49
76	Overlap between Situational and Epistemic Modal Marking	OvSitEpi	0.18	0.26
4	Voicing in Plosives and Fricatives	VoicPF	0.19	0.60
7	Glottalized Consonants	GlotC	0.19	0.41
64	Nominal and Verbal Conjunction	NomVConj	0.19	0.22
126	When’ Clauses	WhenC	0.20	0.59
104	Order of Person Markers on the Verb	PersMV	0.21	0.56
2	Vowel Quality Inventories	Vowel	0.21	0.34
38	Indefinite Articles	IndefArt	0.21	0.29
67	The Future Tense	FutTense	0.21	0.60
85	Order of Adposition and Noun Phrase	AdposNP	0.21	0.44
8	Lateral Consonants	LatC	0.22	0.36
65	Perfective/Imperfective Aspect	PerfImpAsp	0.23	0.57
12	Syllable Structure	SylStr	0.23	0.52
24	Locus of Marking in Possessive Noun Phrases	LmarkPNP	0.24	0.66
109	Applicative Constructions	ApplicativeC	0.24	0.52
83	Order of Object and Verb	OV	0.24	0.53
23	Locus of Marking in the Clause	LmarkC	0.25	0.79
79	Suppletion According to Tense and Aspect	SuppTAsp	0.26	0.71
119	Nominal and Locational Predication	NomLocPred	0.28	0.52
70	The Morphological Imperative	MorphImp	0.29	0.60
1	Consonant Inventories	Cons	0.29	0.45
68	The Perfect	Perfect	0.29	0.73
10	Vowel Nasalization	VowelN	0.30	0.71
107	Passive Constructions	PassiveC	0.32	0.79
6	Uvular Consonants	UvulC	0.34	0.85
113	Symmetric and Asymmetric Standard Negation	SymAsymStNeg	0.35	0.50
30	Number of Genders	NoGen	0.36	0.84
53	Ordinal Numerals	OrdNum	0.37	0.60
50	Asymmetrical Case-Marking	AsymCaseM	0.39	0.85
13	Tone	Tone	0.39	0.57
42	Pronominal and Adnominal Demonstratives	PadDem	0.39	0.69
80	Verbal Number and Suppletion	VnumSupp	0.39	0.81
77	Semantic Distinctions of Evidentiality	SemDistEv	0.40	0.68
27	Reduplication	Redup	0.40	0.56
45	Politeness Distinctions in Pronouns	PolitDPron	0.41	0.52
120	Zero Copula for Predicate Nominals	ZeroCopPredNom	0.41	0.75
108	Antipassive Constructions	AntipassiveC	0.41	0.71
66	The Past Tense	PastTense	0.42	0.61
55	Numeral Classifiers	NumClas	0.44	0.77
57	Position of Pronominal Possessive Affixes	PosProPAff	0.44	0.76
49	Number of Cases	Ncases	0.45	0.92
102	Verbal Person Marking	VpersM	0.45	0.76
118	Predicative Adjectives	PredAdj	0.47	0.83
59	Possessive Classification	PosClas	0.47	0.77
58	Obligatory Possessive Inflection	OlbPosInfl	0.48	0.90
73	The Optative	Optative	0.50	0.87
136	M-T Pronouns	MTPron	0.55	0.82
129	Hand and Arm	HandArm	0.58	0.76
11	Front Rounded Vowels	FrRoundV	0.89	0.98

The features (abbreviated names are transparently based on the full WALS names and as for Figure 4) sorted by the disagreement between methods (IQR). **IQR** (interquartile range) and **Range** (Max - Min) between relative stability ranks as given by all methods.

The IQR varies between 0.08 and 0.89 with a mean of 0.30, and while some features show very little disagreement between methods (such as 87: *Order of Adjective and Noun*, 86: *Order of Genitive and Noun* and 18: *Absence of Common Consonants*), for some (such as 11: *Front Rounded Vowels*, 129: *‘Hand’ and ‘Arm’* and 136: *M-T Pronouns*) the methods disagree almost completely. In order to better understand these IQR values, we computed their expected distribution by randomizing 10,000 times the stability estimates across features and computing the IQR between the methods; the distribution is relatively normal (as judged from the QQ-plot) with a mean of 0.45 and standard deviation of 0.14. For each feature we them compared its IQR to this expected distribution and we found that, after Holm’s correction for multiple testing [62], only feature 87: *Order of Adjective and Noun* has an IQR less than expected (adjusted 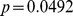), and feature 11: *Front Rounded Vowels* has an IQR much larger than expected (adjusted 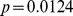). Without multiple testing correction, 24 features have a smaller IQR than expected at an 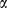-level of 0.05, but only 11: *Front Rounded Vowels* has a larger IQR (see Figure 4 for details). For future research it will be interesting to clarify for individual features why the methods disagree and what these disagreements mean from a theoretical perspective.

## Discussion

Based on our literature survey, we selected seven methods that propose different approaches to defining and quantifying the stability of the structural features of language, generally understood as the inverse of the easiness with which features change value across time, under the influence of various processes. These methods are: **CM**, **CC** and **CR** [43] three related methods which estimate the consistency between the distributional pattern of a feature with the overall distribution of all features using various measures of such consistency; **D** [35] which proposes a fully phylogenetic approach inspired from evolutionary biology; **M** [53], [54] estimating the transition probabilities using pairs of closely related languages; **P**_1_ and **P**_2_ [58] which conceptualize and estimate the borrowability of structural features in contrast to their genealogical stability; and **W** [59] which defines stability as the tendency for sharing between related languages.

The methods **D**, **M** and **W** are all based on the same fundamental genealogic insight, namely that related languages will tend to share the values of stable features (through inheritance from their common ancestor), but these methods still vary widely in their assumptions and implementation. In contrast, **P**_1_ and **P**_2_ look at those features that resist borrowing across genealogical units, while **CM**, **CC** and **CR** focus on those features that show the distributional pattern as expected from the overall pattern when all features are taken together.

If we were to assume a “competing forces” framework such as suggested by Nichols [41], distinguishing between *Inheritance* (vertical transmission from ancestor to descendant language), *Borrowing* (propensity to be acquired by horizontal processes), *Substratum* (persistence from substratum languages), and *Selection* (a bias favouring certain feature values), then each of these methods can be seen as estimating a particular weigthed combination of “forces”. It is beyond the scope of this paper to analyze these combinations (and it is even unclear how these weights could be empirically determined in the absence of pure estimators of the “forces”), but see Table 9 for a subjective attempt based on the description of the methods and the stability estimates they produce (Table 4). Future computational work could simulate different types of features evolving under the influence of known combinations of forces and analyze the stability estimates produced by different methods.

**Table 9:** Nichols’ [41] forces estimated by the seven methods.

Method	Forces			
	Inheritance	Borrowing	Substratum	Selection
CM	+	? +	? −	? ±
CC	+	? +	? −	? ±
CR	+	? +	? −	? ±
D	+++	−	−	+ +
M	+++	−	−	±
P_1_	+ +	− − −	? −	? +
P_2_	+ +	− − −	? −	? +
W	+++	−	−	±

A subjective view on what combination of “forces” each method estimates. Cells represent approximate effects: + (postive effect), − (negative effect), ? (unclear), ± (could be positive or negative).

Probably the most important result of our analysis is that, despite the large conceptual differences between the reviewed methods, they do tend to agree to a large extent. The simplest proposal to explain this agreement is that it is related to an *intrinsic tendency* of some structural features to be more stable than others across language families and geographic areas. Such tendencies are due to multiple factors affecting how features change, including cognitive, articulatory and perceptual biases, and constraints deriving from language use. One important mechanism might be represented by the iterated cultural transmission of language across generations in populations of biased language acquirers and users [63]–[65], and some of these biases might even have a genetic component [66], [67]. From the loadings on the first Principal Component (Tables 5 and 6), the pairwise correlations (Tables 3 and 4) and the distances (Figure 2) between methods, it is clear that while **CM** is discordant (but still agreeing), the other methods contribute relatively equally.

This suggests (i) that this agreement reflects mostly a vertical/genealogical component whereby the stable features’ values tend to be transmitted faithfully to daughter languages, and (ii) that genealogically (vertically) stable features also tend to be stable against contact (horizontal) processes. However, we would first need to rule out other possible explanations, such the pattern of missing data or hidden sampling biases in the considered features, languages, language families and areas. To effectively address these issues we will need to use realistic computer simulations and randomization of the WALS data, as well as, if possible, replications using other typological databases. We will leave this task for future research. Nevertheless, we would argue that, given the differences between the methods included and the fact that each individual publication introducing these methods performed various sanity checks and tests, this agreement does, with a very high probability, tell us something about the stability of structural features and not about contingent sampling and coding biases or dataset coverage.

Second, a clear and reliable distinction seems to cut across conceptual differences between methods. Based on an *a priori* analysis of the methods, we would not have expected that the strongly phylogenetic method **D** and the strongly non-genealogical methods **CC** and **CR** would agree so well. In turn, we would have expected **D** to agree with **W** and **M**, which all include a strong genealogic component. Surprisingly, **W** does not agree very well with **D** and **M**. In effect, the strongest agreement seems to be among the methods **D**, **M**, **CC**, and **CR**. The special status of the borrowability encapsulated by **P_1_** and **P_2_** and the consistency captured by **CM**, **CC** and **CR** also appears in the lower-ranked components of the PCA. The pattern of differences and similarities between methods that our investigation here has uncovered will help clarify not only the various aspects behind the apparently simple concept of structural stability, but also to allow the choice of the most appropriate concept of stability and associated estimation method for the problem at hand.

Third, the identification of globally stable and unstable features (Table 7) as well as those features for which the methods agree or disagree most, will allow a better understanding of language change and evolution and the multifaceted constraints acting on it. However, an important future direction will be represented by the study of structural stability at the level of language family and geographic area. An important first step has recently been made in this direction [36], showing that besides the universal tendencies and idiosyncratic differences between language families, there might be large-scale cross-family patterns in what concerns the stability of structural features.

In this context, it is interesting to note the recent work of Balthasar Bickel [38], which tests the *Family Bias Theory*, whereby “directional biases” attested across multiple families are due to the action of one or more “driving factors” (or “universal pressures”) as opposed to the action of “faithful inheritance”. A directional bias is defined in this context as the skewing of the distribution of a given structural feature’s values in the family’s (or other historical unit’s) languages as detected by a significant 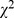 test at 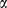-level 0.05 when the *p*-values are computed using permutations (p.2–4). He then adduces several arguments based on the analysis of the WALS database [39] and computer simulations in support of the Family Bias Theory and concludes that “typological distributions are systematically driven by the interaction of faithful inheritance (genealogical stability) with various kinds of external pressure, such as universal principles and areal diffusion trends” (p.17).

Given the results of our analysis, and our discussion of stability in a biological evolutionary context, it seems to us that Bickel’s [38] apparent criticism is in fact agreeing with our findings on a deeper level. More precisely, the “copying fidelity” of a structural feature across generations and language splits is certainly a component of the feature’s stability but not the only one, as external pressures (“driving factors”), generated by language-internal or by the larger cultural, biological and ecological context, also play an important role in shaping the distribution of linguistic diversity. Linguists have been aware for a long time that languages have historical and evolutionary inertia and that different languages and language groups present different affordances and opportunities for language change, all these interacting in a complex manner with feature-specific properties in shaping their temporal stability.

In conclusion, this analysis represents an important step towards a better understanding of language as a complex evolutionary system, and it strongly suggests that structural stability shows a clear universal tendency for some features to be more stable than others, but that this apparently simple concept of stability is in fact very complex.

## Supporting Information

Methods S1 **Elena Maslova’s estimation of transition probabilities.** Here we present the detailed derivation of Elena Maslova’s (denoted in this paper as method **M**) estimation of transition probabilities starting from first prinsiples and its application to the WALS data.
(PDF)

Script S1 **The R implementation of Maslova’s method.** This is the R script implementing our derivation of Maslova’s method (detailed in **Methods S1**) for the WALS database, released under a GPL v3 license.
(R) Click here for additional data file.

Dataset S1 **The version of WALS dataset used in the paper.** This dataset (released under an Attribution-NonCommercial-NoDerivs 2.0 Germany (CC BY-NC-ND 2.0) license) is the actual version of WALS used in our paper, included here for maximum reproductibility of the reported results.
(GZ) Click here for additional data file.
