# Supplementary material for: Some Structural Aspects of Language Are More Stable than Others: A Comparison of Seven Methods
Source: PLoS One. 2013 Jan 28;8(1):e55009. doi: 10.1371/journal.pone.0055009 (PMC3557264; doi:10.1371/journal.pone.0055009)
Supplement: Methods S1 — Elena Maslova’s estimation of transition probabilities. Here we present the detailed derivation of Elena Maslova’s (denoted in this paper as method M) estimation of transition probabilities starting from first prinsiples and its application to the WALS data. (PDF) [file pone.0055009.s001.pdf]

Some Structural Aspects of Language are More Stable than Others: A Comparison of Seven Methods

– Supporting Online Information –

Dan Dediu[1,2,*], Michael Cysouw[3]
1 Language and Genetics Department, Max Planck Institute for Psycholinguistics, Nijmegen, The Netherlands
2 Donders Institute for Brain, Cognition and Behaviour, Nijmegen, The Netherlands
3 Forschungszentrum Deutscher Sprachatlas, Philipps Universität Marburg, Germany
∗ E-mail: Dan.Dediu@mpi.nl

Methods S1. Elena Maslova's estimation of transition probabilities

Introduction

We present here in detail the derivation of Elena Maslova's estimation of transition probabilities (denoted **M** in our paper), as well as some possible extensions. A more general introduction can be found in [1]. This presentation is based on [2–5] and on personal communication between Cysouw and Maslova in 2006. The technical details of Maslova's proposals are not easily extractable from her publications, and because there is still no detailed description from her own hand we decided to publish our rendition of her ideas here. As is discussed in the main paper, Maslova's approach can be seen as a simplified formalisation of the method used in [6] (denoted **D** in our paper) and empirically strongly correlated to it. Considering that Maslova's approach is much easier to handle, as both the empirical prerequisites (i.e. only pairs of related languages are necessary) and the mathematical calculations (i.e. only some quadratic equations have to be solved) are simpler, we propose that Maslova's approach can be used to quickly obtain approximate estimates of transition probabilities.

Assumptions

Consider a typology of languages for a particular feature. To estimate the *transition probabilities*, we will restrict ourselves here to features with two possible types only, A and B. Languages have to be either of type A or of type B, nothing else is allowed (but see Section for extensions of this restriction). This restriction to binary features immediately implies that the fraction of languages of type A is the complement of the fraction of languages of type B. Or, stated in terms of probabilities:

$$P(A) + P(B) = 1 \tag{1}$$

where $P(A)$ and $P(B)$ are the fraction of languages of type A and B, respectively.

Within a period of time t, ranging from t_0 to t_1, we attempt to estimate the probability that languages will change from A to B, or vice versa from B to A. We do not make any assumptions concerning the *causes* of such changes: they can be due to internal developments within the language or to external influences (such as contact with another languages); from the current method's point of view these distinctions are irrelevant.

Let p_{AB} be the probability that a language of type A changes to type B within the timeframe t. Likewise, let p_{BA} be the probability of a change from B to A. These two probabilities are independent of each other. The complement of p_{AB}, viz. $1 - p_{AB}$, can be interpreted as the probability that a language of type A does not change to type B within the timeframe t. Likewise, the complement of p_{BA}, viz. $1 - p_{BA}$, is the probability that a language of type B does not change to A.

Of course, within the timeframe t a language might change from A to B and back to A (A $\rightarrow$ B $\rightarrow$ A), in which case it would be included in the $1 - p_{AB}$ because, at the end of t, it would still be of type A. This applies to any odd number of changes (A $\rightarrow$ B $\rightarrow \ldots \rightarrow$ B $\rightarrow$ A) and shows that (i) t must be short enough such that such *reversals* to the original value are not too frequent, and (ii) that this method is unable to account for reversals, as opposed to more advanced likelihood and Bayesian phylogenetic methods [7, 8]. The necessary assumption that the period t is short is a limiting factor on Maslova's approach. However, in practice there is not much accepted knowledge about deep phylogenies in linguistics anyway, so empirically linguists will mostly work within groups of closely related languages, or even with variation between dialectal variants.

Assuming that these transition probabilities *remain the same* over longer periods of time, then it is possible to predict the stable distributions of the types A and B, i.e. the situation in which the fraction of languages of type A and B does not change: $P_{t_1}(A) = P_{t_0}(A)$ and $P_{t_1}(B) = P_{t_0}(B)$. Such a stable situation does not mean that there are no changes anymore; it means that the number of changes cancel out against each other. The crucial assumption that the transition probabilities themselves remain stable is of course far from proven. It might very well be the case that even these probabilities have changed over time. Still, the assumption of universal transition probabilities represents a step forward from the common practice of linguistic typology to assume universal empirical frequencies [1].

Basic implications

Concretely, in a stable distribution P_S there are equally many languages changing from A to B as there are languages changing from B to A within a particular period of time, so:

$$P_S(A) \cdot p_{AB} = P_S(B) \cdot p_{BA} \tag{2}$$

Using (1) and (2), the fractions of languages of type A and type B in the stable distribution can be predicted from the transition probabilities:

$$\begin{aligned} P_S(A) &= \frac{p_{BA}}{p_{AB} + p_{BA}} \\ P_S(B) &= \frac{p_{AB}}{p_{AB} + p_{BA}} \end{aligned} \tag{3}$$

Further, the complement of the average of the transition probabilities, i.e. $1 - \frac{p_{AB}+p_{BA}}{2}$ can be interpreted as *stability*, i.e. the probability that there will be no change in a particular period. A high value indicates that few languages will change, which means that the characteristic is very stable. Conversely, a low average probability is indicative that a characteristic is highly unstable. However, as discussed above, this interpretation crucially hangs on the absence of hidden reversals within the timeframe t and thus on the careful choice of t.

Finally, the expected fraction of languages of type A and type B at the end of a period t can be predicted from the fractions at the start of the period. Namely, the languages of type A at the end of the period $P_{t_1}(A)$ will consist of those languages that were of type A at the start of the period $P_{t_0}(A)$ which did not change to B, i.e. with probability of $1 - p_{AB}$, together with those languages that were of type B at the start of the period $P_{t_0}(B)$ which did change to A, i.e. with probability p_{BA}. The same holds reversely for $P_{t_1}(B)$.

$$\begin{cases} P_{t_1}(A) = P_{t_0}(A) \cdot (1 - p_{AB}) + P_{t_0}(B) \cdot p_{BA} \\ P_{t_1}(B) = P_{t_0}(A) \cdot p_{AB} + P_{t_0}(B) \cdot (1 - p_{BA}) \end{cases} \tag{4}$$

Using (1), these equations can be inverted to expressions of the fractions at the beginning of the period in terms of the fractions at the end of the period (we will need this in the next section for the estimation

of the transition probabilities), e.g. for type A:

$$
\begin{aligned}
P_{t_1}(A) &= P_{t_0}(A) \cdot (1 - p_{AB}) + P_{t_0}(B) \cdot p_{BA} \\
P_{t_1}(A) &= P_{t_0}(A) \cdot (1 - p_{AB}) + [1 - P_{t_0}(A)] \cdot p_{BA} \\
P_{t_1}(A) &= P_{t_0}(A)(1 - p_{AB} - p_{BA}) + p_{BA} \\
\frac{P_{t_1}(A) - p_{BA}}{1 - p_{AB} - p_{BA}} &= P_{t_0}(A) \qquad (5)
\end{aligned}
$$

Doing this likewise for type B results in:

$$
\begin{cases}
P_{t_0}(A) = \frac{P_{t_1}(A) - p_{BA}}{1 - p_{AB} - p_{BA}} \\
P_{t_0}(B) = \frac{P_{t_1}(B) - p_{AB}}{1 - p_{AB} - p_{BA}}
\end{cases} \qquad (6)
$$

Estimating transition probabilities

Linguists are often highly confident that certain languages are related, without necessarily being able to reach agreement on the details of the internal subgrouping of such a genealogical unit. Taking advantage of this empirical situation, we will only assume that (see also the discussion above):

1. there is a distinction between pairs of related vs. non-related languages (i.e. there are no detailed genealogical trees assumed)

2. the time-depth of split-up of related languages is relatively small, so that it is likely that there has maximally been one single change of type per language in that period (no reversals) and that there is a low probability that all related languages have changed.

3. all pairs of related languages have approximately the same time depth. In practice we used the genus-level as described in WALS as the maximum divergence time depth.

Given a sample of *pairs* of such related languages we can estimate the transition probabilities and the stability of the concerned features (see Section) – the method we applied in this paper, and which amounts to the original proposal from Maslova. However, two interesting extensions are also possible: (a) we could look at *groups of three* of such related languages (see Section) and (b) we could add to the sample of related pairs a third, non-related language but which is geographically close, allowing us to estimate the transition probabilities including borrowing events into the model (see Section). Interestingly, the resulting formulas to estimate the transition probabilities in these cases only differ by a constant factor.

Using genealogically closely related pairs

Given a pair of closely related languages, the method assumes that they both shared the same type at the start t_0 of the period t. Either both languages are of type A, with probability $P_{t_0}(A)$, or both are of type B, with probability $P_{t_0}(B)$. Some changes might happen (or not) during the period t, resulting in a particular probability that both languages are still identical at the end t_1 of the period t. This probability is called $P_{t_1}(identical)$. This probability is the sum of four possible histories: either both languages started off as type A and both did not change ($AA_{t_0} \rightarrow AA_{t_1}$); or both started off as type A and both changed to B ($AA_{t_0} \rightarrow BB_{t_1}$); or both started off as type B and both did not change ($BB_{t_0} \rightarrow BB_{t_1}$); or, finally, both started of as type B and both changed to A ($BB_{t_0} \rightarrow AA_{t_1}$). Note that the assumption of a short time-span t leads to the further assumption that the number of pairs that did not change ($AA_{t_0} \rightarrow AA_{t_1}$ and $BB_{t_0} \rightarrow BB_{t_1}$) will be larger than the number of pairs that changed completely ($AA_{t_0} \rightarrow BB_{t_1}$ and $BB_{t_0} \rightarrow AA_{t_1}$). This assumption will become important in the solving of

the equations (see Section). Thus, the probability of pairs of languages being identical in a synchronic empirical collection of pairs can be expressed as:

$$\begin{aligned} P_{t_1}(identical) = P_{t_1}(I) = \quad & P_{t_0}(A) \cdot (1 - p_{AB})^2 + P_{t_0}(A) \cdot p_{AB}^2 \\ & + P_{t_0}(B) \cdot (1 - p_{BA})^2 + P_{t_0}(B) \cdot p_{BA}^2 \end{aligned} \tag{7}$$

Using (1), this can be reformulated as an equation relating the fraction of identical pairs at the end of the period $P_{t_1}(I)$ and the fraction of languages of type A at the beginning of the period $P_{t_0}(A)$:

$$\begin{aligned} P_{t_1}(I) &= P_{t_0}(A) \cdot \left[(1 - p_{AB})^2 + p_{AB}^2\right] + (1 - P_{t_0}(A)) \cdot \left[(1 - p_{BA})^2 + p_{BA}^2\right] \\ &= P_{t_0}(A) \cdot \left[(1 - p_{AB})^2 + p_{AB}^2 - (1 - p_{BA})^2 - p_{BA}^2\right] + (1 - p_{BA})^2 + p_{BA}^2 \end{aligned}$$

Using (6), the fraction of type A at the beginning of the period can be expressed in terms of the fraction of type A at the end of the period. Thus, both $P(I)$ and $P(A)$ in the equation are expressed at the same point in time, reducing the necessity for a subscript for time:

$$P(I) = \frac{P(A) - p_{BA}}{1 - p_{AB} - p_{BA}} \cdot \left[(1 - p_{AB})^2 + p_{AB}^2 - (1 - p_{BA})^2 - p_{BA}^2\right] + (1 - p_{BA})^2 + p_{BA}^2$$

This simplifies to

$$P(I) = P(A) \cdot 2(p_{BA} - p_{AB}) - 2p_{BA}(1 - p_{AB}) + 1$$

or, by defining $P(D)$ as the complement of $P(I)$, i.e. $P(D) = 1 - P(I)$, this becomes

$$P(D) = 1 - P(I) = P(A) \cdot 2(p_{AB} - p_{BA}) + 2p_{BA}(1 - p_{AB}) \tag{8}$$

$P(D)$ is the frequency with which the languages within the pair are different. So, there should be a *linear dependency* between the frequency of pairs of languages being different, $P(D)$, and the frequency of languages of type A, $P(A)$, of the form $P(D) = 2\alpha \cdot P(A) + 2\beta$, with $\alpha = p_{AB} - p_{BA}$ and $\beta = p_{BA}(1 - p_{AB})$. By empirically measuring $P(D)$ and $P(A)$ and by estimating the coefficients α and β of the linear dependency, it is possible to estimate the transition probabilities (see Section for the practical details).

Note that it might seem to be even more interesting to consider a less constrained model, starting with two languages of any pair of types, AA, AB, BA, or BB. This would result in the following equation for $P(I)$:

$$\begin{aligned} P_{t_1}(I) = \quad & P_{t_0}(A)^2 \left[(1 - p_{AB})^2 + p_{AB}^2\right] \\ & + P_{t_0}(B)^2 \left[(1 - p_{BA})^2 + p_{BA}^2\right] \\ & + 2 \cdot P_{t_0}(A) \cdot P_{t_0}(B) \left[p_{AB}(1 - p_{BA}) + p_{BA}(1 - p_{AB}\right] \end{aligned}$$

However, after performing the same algebra as above, all transition probabilities factor out, leaving just the evidently true, but useless, equation

$$P(I) = 2 \cdot P(A)^2 - 2 \cdot P(A) + 1 = P(A)^2 + P(B)^2$$

Using genealogically closely related triples

There are various different setting that can be used to estimate the transition probabilities. However, most of them quickly become rather complex. The algebra of the following two settings also nicely reduces to a manageable model, being only slightly different from the previous one. These settings were not considered by Maslova herself, but added by the present authors.

Instead of looking at pairs of languages, one might also look at groups of three closely related languages. In that case the probability that all three languages are identical consists of four different possible histories ($AAA_{t_0} \rightarrow AAA_{t_1}$, $AAA_{t_0} \rightarrow BBB_{t_1}$, $BBB_{t_0} \rightarrow BBB_{t_1}$ and $BBB_{t_0} \rightarrow AAA_{t_1}$). This results in an equation very similar to (7):

$$\begin{aligned} P_{t_1}(I) = \; & P_{t_0}(A) \cdot (1 - p_{AB})^3 + P_{t_0}(A) \cdot p_{AB}^3 \\ & + P_{t_0}(B) \cdot (1 - p_{BA})^3 + P_{t_0}(B) \cdot p_{BA}^3 \end{aligned}$$

Performing the same algebra as in the previous section, this leads to:

$$P(D) = P(A) \cdot 3(p_{AB} - p_{BA}) + 3p_{BA}(1 - p_{AB}) \tag{9}$$

i.e. exactly the same formula as in (8), though with a constant 3 instead of 2. Note that this *does not* generalize to larger groups, i.e. for groups with five languages it does not work to replace the constant with a 5. All groups higher than three languages lead to much more complex algebra and thus are not usable as a quick approximation (which is the goal of the present method).

Using geographically close, but genealogically unrelated pairs

In this extension of Maslova's approach we again consider two closely related languages, but now we add a third non-related language that is geographically close to one (and only one) of the two related languages. We are interested in situations where the two related languages are of a different type, but the two non-related yet geographically close languages are of the same type. Such a situation is typically interpreted as the result of contact-induced change in one of the geographically close languages. However, there are various histories possible that lead to this setting.

We assume, as before, that at the start t_0 of the period t the two related languages are of the same type, so either both are of type A, with probability $P_{t_0}(A)$, or both are of type B, with probability $P_{t_0}(B)$. The non-related third language can also be of either type (with the same probabilities), so there are four possible start settings: AA-A, AA-B, BB-A, and BB-B (the non-related, but geographically close language is shown separated by a dash). We are interested in end situations in which the two related languages are of different types, but the two geographically close language are of the same type: AB-B and BA-A. The probability for any of these situations to occur will be denoted $P(C)$, where the 'C' mnemonically stands for 'convergence'.

From each starting situation it is possible to arrive at both end situations, given the right changes. For example, to get from AA-A to AB-B requires two languages to change from A to B and one language to not change from type A. Writing out all eight such possibilities gives the following unwieldy formula:

$$\begin{aligned} P_{t_1}(C) = \; & P_{t_0}(A)^2 \left[(1 - p_{AB})^2 p_{AB} + (1 - p_{AB}) p_{AB}^2\right] \\ & + P_{t_0}(B)^2 \left[(1 - p_{BA})^2 p_{BA} + (1 - p_{BA}) p_{BA}^2\right] \\ & + P_{t_0}(A) \cdot P_{t_0}(B) \left[p_{AB} p_{BA}(1 - p_{AB}) + p_{BA}(1 - p_{AB})(1 - p_{BA})\right] \\ & + P_{t_0}(A) \cdot P_{t_0}(B) \left[p_{AB} p_{BA}(1 - p_{BA}) + p_{AB}(1 - p_{AB})(1 - p_{BA})\right] \end{aligned}$$

However, this immediately reduces to

$$\begin{aligned} P_{t_1}(C) = \; & P_{t_0}(A)^2 \left[(1 - p_{AB}) p_{AB}\right] \\ & + P_{t_0}(B)^2 \left[(1 - p_{BA}) p_{BA}\right] \\ & + P_{t_0}(A) \cdot P_{t_0}(B) \left[(1 - p_{AB}) p_{AB}\right] \\ & + P_{t_0}(A) \cdot P_{t_0}(B) \left[(1 - p_{BA}) p_{BA}\right] \end{aligned}$$

Combining terms, and using the complementarity of $P(A)$ and $P(B)$, this reduces to

$$P_{t_1}(C) = P_{t_0}(A)\left[(1-p_{AB})p_{AB} - (1-p_{BA})p_{BA}\right] + (1-p_{BA})p_{BA}$$

Using (6), the probability $P_{t_0}(A)$ can be expressed by using only the probability $P_{t_1}(A)$, so the time-subscripts are identical and can thus be left out:

$$P(C) = \frac{P(A) - p_{BA}}{1 - p_{AB} - p_{BA}} \cdot \left[(1-p_{AB})p_{AB} - (1-p_{BA})p_{BA}\right] + (1-p_{BA})p_{BA}$$

which reduces nicely to

$$P(C) = P(A) \cdot (p_{AB} - p_{BA}) + p_{BA}(1 - p_{BA}) \qquad (10)$$

So, again there is the same *linear dependency* between the probability that the two unrelated languages are identical while the two related languages are different, $P(C)$, and the empirical probability of languages being of type A, $P(A)$. The only difference between (10) and the earlier results in (8) and (9) is the constant. By estimating the coefficients, it is possible to estimate the transition probabilities, and from that the stable distribution and the stability of the feature.

Empirically estimating the transition probabilities using *WALS*

In the main part of this paper, we applied the method described in Section to the data of the *World Atlas of Language Structures* (WALS) [9] in order to obtain estimates of the stability of the structural features of language covered in this database. The actual `R` code (released under a GPL v3 license) is given below.

For *any given feature* in WALS, F with $n \geqslant 2$ values $V_1, \ldots V_n$, we estimated separately the transition probabilities for each of its values, V_i such that with the previous notations A is V_i and B represents all other values except for V_i. Thus, for *each value* V_i we estimated the transition probabilities from V_i to any other possible value, p_{ABi}.

The basic idea is the following. For a specific value A we select pairs of languages of the same genus from WALS. All those pairs are separated into two different samples, for convenience called sample 1 and sample 2 here. For each of these samples, we count how many pairs are not identical (so, one of the two languages has value A, the other has not value A). The proportions of different pairs for the two samples are $P(D_1)$ and $P(D_2)$. Further, for both samples we count the number of languages that have value A. The proportions of value for the two samples are $P(A_1)$ and $P(A_2)$. From equation (8) we then have:

$$\begin{cases} P(D_1) = 2\alpha P(A_1) + 2\beta \\ P(D_2) = 2\alpha P(A_2) + 2\beta \end{cases} \text{with} \begin{cases} \alpha = p_{AB} - p_{BA} \\ \beta = p_{BA}(1 - p_{AB}) \end{cases} \qquad (11)$$

From the first two equations in (11) we can derive:

$$\alpha = \frac{1}{2} \cdot \frac{P(D_1) - P(D_2)}{P(A_1) - P(A_2)}$$

$$\beta = \frac{1}{2} \cdot \frac{P(A_1)P(D_2) - P(A_2)P(D_1)}{P(A_1) - P(A_2)}$$

And from the second two equations in (11) we can derive:

$$p_{AB} = \frac{1 + \alpha \pm \sqrt{(1-\alpha)^2 - 4\beta}}{2}$$

$$p_{BA} = \frac{1 - \alpha \pm \sqrt{(1-\alpha)^2 - 4\beta}}{2}$$

By filling in the four empirical estimates for $P(D_1)$, $P(D_2)$, $P(A_1)$ and $P(A_2)$ we can thus directly derive estimates for p_{AB} and p_{BA}. However, note that there are actually two solutions for p_{AB} and p_{BA}, one with lower transition probabilities (the 'minus' variant) and one with higher transition probabilities (the 'plus' variant), with $p^-_{AB} = 1 - p^+_{BA}$ and vice versa. The interpretation of these two solutions can be understood from looking back at equation (7). The 'minus' solution represents the situation that the number of pairs that did not change ($AA_{t_0} \rightarrow AA_{t_1}$ and $BB_{t_0} \rightarrow BB_{t_1}$) is larger than the number of pairs that changed completely ($AA_{t_0} \rightarrow BB_{t_1}$ and $BB_{t_0} \rightarrow AA_{t_1}$). As was discussed in Section , this is the fitting interpretation to the assumption that the time period t is small. We will therfore use the 'minus' solution here.

Instead of using just two samples of pairs, as illustrated above, it is also possible to select many different samples. In fact, in order to estimate α and β we used multiple sets of $P(A)$ and $P(D)$ for the same value. Our way of obtaining these multiple sets is by randomly subsampling the set of all available genera. For example, using WALS for feature 10 (*Vowel Nasalization*) and its value 1 ("Contrast present"), we have a total of 26 genera with enough data for this feature[1] We created 50 random subsets of 13 genera and for each such subset we computed the $P(D)$ and $P(A)$, as exemplified in Table 1.

Table 1. Example $P(A)$ and $P(D)$ for feature 10 (*Vowel Nasalization*) in WALS. Each subset shown is composed of 13 random genera (here given by their alphabetical index, thus 1 represents *Adamawa-Ubangian*). As an example, we only show the first 10 subsets generated in one particular run.

**Subset**	$P(A)$	$P(D)$
9, 11, 13, 18, 26, 10, 5, 23, 17, 7, 20, 25, 15	0.23	0.15
17, 2, 7, 8, 5, 26, 20, 22, 18, 23, 4, 25, 1	0.27	0.23
18, 19, 14, 11, 6, 25, 13, 17, 2, 22, 3, 12, 10	0.31	0.15
4, 5, 26, 2, 16, 24, 14, 10, 12, 25, 3, 1, 20	0.35	0.23
12, 9, 20, 11, 5, 24, 23, 22, 7, 1, 17, 6, 25	0.46	0.31
19, 8, 11, 14, 22, 21, 17, 6, 26, 16, 25, 2, 15	0.31	0.31
5, 1, 16, 6, 18, 25, 15, 8, 19, 13, 9, 23, 2	0.12	0.08
24, 14, 9, 1, 4, 11, 6, 16, 23, 10, 18, 20, 19	0.38	0.15
18, 26, 6, 4, 14, 12, 1, 13, 11, 8, 25, 20, 24	0.38	0.31
13, 25, 22, 3, 8, 14, 12, 4, 9, 21, 20, 2, 16	0.27	0.38

If we then regress $P(D)$ to $P(A)$ we obtain estimates of α and β as the coefficients of this regression. In this example $\alpha = 0.08$ (std. error 0.17) and $\beta = 0.17$ (std. error 0.05). The error values show that these estimates are not completely random, though it should be noted that the errors are substantial. The estimates are thus to be interpreted with care. Still, proceeding with these estimated parameters we can estimate $p_{AB} = 0.33$ and $p_{BA} = 0.25$. Using the formula in (3) this implies that the stable distribution of $P_S(A) = 0.43$. Note that the actual frequency of vowel nasalization in WALS is only 26%, indicating that the current distribution is not in its stable state, and that there probably is influence from historical coincidences on the current world-wide distribution of vowel nasalisation. The stability of this characteristic is rather high, so languages do not seem to change too often, making it even more probable that the current distribution shows signs of historical events.

Further, we computed the stability of the feature F by taking the *weighted average* of the stability of each of its values V_i (defined as $1 - p_{ABi}$), where the weights are represented by the relative frequencies

[1]The 26 genera are: *Adamawa-Ubangian*, *Algonquian*, *Athapaskan*, *Bantoid*, *Biu-Mandara*, *Bodic*, *Bongo-Bagirmi*, *Cariban*, *Eskimo-Aleut*, *Germanic*, *Gur*, *Kam-Tai*, *Kuki-Chin-Naga*, *Kwa*, *Madang*, *Northern Atlantic*, *Nupoid*, *Oceanic*, *Pama-Nyungan*, *Romance*, *Semitic*, *Southern Atlantic*, *Sundic*, *Tupi-Guaraní*, *Western Mande*, and *Yuman.*

of the feature values:

$$S(F) = \frac{1}{n}\sum_{i=1}^{n}(1 - p_{ABi})P_i \tag{12}$$

where P_i is the frequency of value V_i relative to all the possible values of feature F. Thus, the stability of more frequent values have a bigger influence on estimating the frequency of the whole feature's stability $S(F)$.

The R code

This section contains the R code (released under GPL v3 and also reproduced in **Script S1**) implementing the estimation of stability (used in this paper) based on Elena Maslova's method (as described above). Please note that depending on the version of the WALS database used results might differ slightly from the ones reported here, but for maximum reproductibility we also included the version of the WALS dataset we used as **Dataset S1** (released under an Attribution-NonCommercial-NoDerivs 2.0 Germany (CC BY-NC-ND 2.0) license [http://creativecommons.org/licenses/by-nc-nd/2.0/de/deed.en]; see the included ReadMe.txt for more details). Also, estimating the transition probabilities (lines 207-212) is very CPU-intensive. The symbols ↘ and ↳ mark the points where a long line breaks to fit the page.

```
1 ##############################################################################
  #     Estimate and generalize Elena Maslova's "transition probabilities"
3 #
  #     Copyright (C) 2008-2012  Michael Cysouw & Dan Dediu
5 #
  #     This program is free software: you can redistribute it and/or modify
7 #     it under the terms of the GNU General Public License as published by
  #     the Free Software Foundation, either version 3 of the License, or
9 #     (at your option) any later version.
  #
11 #     This program is distributed in the hope that it will be useful,
  #     but WITHOUT ANY WARRANTY; without even the implied warranty of
13 #     MERCHANTABILITY or FITNESS FOR A PARTICULAR PURPOSE.  See the
  #     GNU General Public License for more details.
15 #
  #     You should have received a copy of the GNU General Public License
17 #     along with this program.  If not, see <http://www.gnu.org/licenses/>.
  #
19 ##############################################################################

21 # The working path:
  working.path <- "./";
23
  # Load the original WALS data as used in Dediu, D. & Cysouw, M. Some Structural ↘
    ↳ Aspects of Language are More Stable than Others: A Comparison of Seven Methods. ( ↘
    ↳ please make sure you have extracted it here):
25 source( paste( working.path, "/WALS_R/wals.r", sep="" ), chdir=TRUE);
  ## Please note that currently the WALS data is released in a slightly different format ↘
    ↳ (see http://wals.info/export) and in order to import it you need to use instead ↘
    ↳ something of the form:
27 ## but we did not test the script with this new format and it might require some ↘
    ↳ tweaking.
  #langs <- read.table("languages.tab",sep="\t",header=T)
29 #mat <- read.table("datapoints.tab",sep="\t",header=T,row.names=1)

31

33 # Select genera that have more than one language coded for a feature
  # For example, "length(getgenera(83))" gives "197"
```

```r
# Constrain by macroarea (if != NA) and/or family (if != NA):
getgenera <- function( feature , macroarea=NA, lgfamily=NA )
{
  # Select those languages in the given macroarea (if any):
  langs <- names( na.omit( mat[,feature] ) );
  if( !is.na( macroarea ) )
  {
    langs <- langs[ lgs$macroarea[ rownames(lgs) %in% langs ] == macroarea ];
  }
  if( !is.na( lgfamily ) )
  {
    langs <- langs[ lgs$family[ rownames(lgs) %in% langs ] == lgfamily ];
  }

  g <- listGenera( codes=langs ) # list all genera
  if( !is.na( macroarea ) & !is.na( lgfamily ) ) # both constraints
  {
    count <- sapply( g, function(x){ length( na.omit( mat[ rownames( lgs[ lgs$genus==x & lgs$macroarea == macroarea & lgs$family == lgfamily , ] ), feature ] ) ) } ) # get genera that are coded more than once
  } else if( !is.na( macroarea ) ) # only macroarea constraint
  {
    count <- sapply( g, function(x){ length( na.omit( mat[ rownames( lgs[lgs$genus==x & lgs$macroarea == macroarea , ] ), feature ] ) ) } ) # get genera that are coded more than once
  } else if( !is.na( lgfamily ) ) # only faimly constraint
  {
    count <- sapply( g, function(x){ length( na.omit( mat[ rownames( lgs[ lgs$genus==x & lgs$family == lgfamily , ] ), feature ] ) ) } ) # get genera that are coded more than once
  } else # no constraints at all:
  {
    count<-sapply(g,function(x){length(na.omit(mat[rownames(lgs[lgs$genus==x,]),feature]))}) # get genera that are coded more than once
  }
  return( g[ count>1 ] )
}

# Get estimates for P(D) and P(A) for a set of genera g.
# First, select a pair of languages from each genus, then compute P(D) and P(A).
# The value can consist either of a single value (A), in which case all the others are considered as (non-A); a list of values (A) and all the others are considered (non-A); or as a list of values (A) and the non.values contains the list of non-A values, with all the others ignored [not yet implemented]
getprobs <- function( g, feature , value , macroarea=NA, lgfamily=NA, non.value=NA )
{
  # Get a random pair of languages from the same genus, for each genus:
  if( !is.na( macroarea ) & !is.na( lgfamily ) ) # Both constraints:
  {
    pairs <- lapply( g, function(x){ sample( na.omit( mat[ rownames( lgs[ lgs$genus==x & lgs$macroarea==macroarea & lgs$family==lgfamily , ] ), feature ] ), 2 ) } )
  } else if( !is.na( macroarea ) ) # only macorarea constraint:
  {
    pairs <- lapply( g, function(x){ sample( na.omit( mat[ rownames( lgs[ lgs$genus==x & lgs$macroarea==macroarea , ] ), feature ] ), 2 ) } )
  } else if( !is.na( lgfamily ) ) # only faimly constraint:
  {
    pairs <- lapply( g, function(x){ sample( na.omit( mat[ rownames( lgs[ lgs$genus==x & lgs$family==lgfamily , ] ), feature ] ), 2 ) } )
  } else # no contraints:
  {
```

```
    pairs <- lapply( g, function(x){ sample( na.omit( mat[ rownames( lgs[lgs$genus==x ,]), feature ] ), 2 ) } )
  }

  # The pA and pD probabilities:
  pA <- NA;
  pD <- NA;

  # Compute pD & pA for these pairs:
  if( is.na(non.value) )
  {
    pD <- 1 - ( sum( as.numeric( lapply( pairs, function(x){ ( (x[1] %in% value) & (x[2] %in% value) ) | ( !(x[1] %in% value) & !(x[2] %in% value) ) } ) ) ) / length( pairs ) );
    pA <- sum( as.matrix( as.data.frame( lapply( pairs, function(x){ x %in% value } ) ) ) ) / ( 2 * length( pairs ) );
  } else
  {
    stop( "No yet implemented!\n" );
  }
  return( data.frame( "pA"=pA, "pD"=pD ) );
}

# Get estimates for P(D) and P(A) multiple times, and put the results in a table:
getcounts <- function( feature, value, cases=50, samplesize=.5, macroarea=NA, lgfamily=NA, non.value=NA )
{
  result <- data.frame( "pA"=numeric(cases), "pD"=numeric(cases) );
  g <- getgenera( feature, macroarea, lgfamily );

  for( i in 1:cases )
  {
    s <- sample( length(g), floor( samplesize*length(g) ) );
    result[i,] <- getprobs( g[s], feature, value, macroarea, lgfamily, non.value );
  }
  return( result );
}

# Compute the transition probabilities from the output of "getcounts", ie. from the table with the values of P(D) and P(A):
estimates <- function( test, no.simulations=1000, return.summaries=TRUE )
{
  # Regress pD an pA:
  l <- lm( pD ~ pA, data=test ); # The regression goodness was tested before using Pearson's r, so don't do it again here!

  # Get the estimated regression coefficients: they have a mean and a standard distribution:
  l.summary <- summary( l );
  if( nrow( l.summary$coefficients ) < 2 )
  {
    a.estimate <- NA;
    a.sd <- NA;
    b.estimate <- NA;
    b.sd <- NA;
  } else
  {
    a.estimate <- as.numeric( l.summary$coefficients[2,1] );
    a.sd <- as.numeric( l.summary$coefficients[2,2] );
    b.estimate <- as.numeric( l.summary$coefficients[1,1] );
    b.sd <- as.numeric( l.summary$coefficients[1,2] );
  }
```

```
  # Given the complexity of the formaulas for pAB and pBA, I cannot derive a formula for their sd, so simulate it:
  a.simulated <- rnorm( no.simulations, mean=a.estimate, sd=a.sd)  / 2;
  b.simulated <- rnorm( no.simulations, mean=b.estimate, sd=b.sd)  / 2;

  # And compute the probabilities:
  pAB.simulated <-  (1 + a.simulated - sqrt((1 - a.simulated)^2 - 4*b.simulated))/2;
  pBA.simulated <-  (1 - a.simulated - sqrt((1 - a.simulated)^2 - 4*b.simulated))/2;
  stableA.simulated   <- pBA.simulated /  (pAB.simulated + pBA.simulated);
  stability.simulated <- 1 -  ((pAB.simulated + pBA.simulated)/2);

  # get correlations for estimates
  correlation <- cor.test( test[,1], test[,2]  )

  # Return the results:
  if( return.summaries == TRUE )
  {
    return( list(
                    pApD.cor = as.numeric(correlation$estimate),
                    pApD.sig = correlation$p.value,
                    pAB.mean = mean( pAB.simulated, na.rm=TRUE ),
                    pBA.mean = mean( pBA.simulated, na.rm=TRUE ),
                    stableA.mean = mean( stableA.simulated, na.rm=TRUE ),
                    stability.mean = mean( stability.simulated, na.rm=TRUE ),
                    pAB.sd = ifelse( sum(  !is.na( pAB.simulated )  ) <= 1, NA, sd( pAB.simulated, na.rm=TRUE )  ),
                    pBA.sd = ifelse( sum(  !is.na( pBA.simulated )  ) <= 1, NA, sd( pBA.simulated, na.rm=TRUE )  ),
                    stableA.sd = ifelse ( sum(  !is.na( stableA.simulated )  ) <= 1, NA, sd( stableA.simulated, na.rm=TRUE )  ),
                    stability.sd = ifelse( sum(  !is.na( stability.simulated )  ) <= 1, NA, sd( stability.simulated, na.rm=TRUE )  )
                  ) );
  } else
  {
    return ( data.frame( pAB=pAB.simulated,   pBA=pBA.simulated,     stableA=stableA.simulated,     stability=stability.simulated )  );
  }
}

# Compute the observed frequency of the feature's value in the given macroarea and language family:
observed.freq <- function( feature, value, macroarea=NA, lgfamily=NA, non.value=NA )
{
  # Select those languages in the given macroarea (if any):
  langs <- names( na.omit( mat[  , feature ]  )  );
  if(  !is.na( macroarea )  )
  {
    langs <- langs[ lgs$macroarea[ rownames(lgs)  %in% langs ]  == macroarea ];
  }
  if(  !is.na( lgfamily )  )
  {
    langs <- langs[ lgs$family[ rownames(lgs)  %in% langs ]  == lgfamily ];
  }

  values <- na.omit( mat[ rownames(lgs)  %in% langs,feature ]  );
  obs.freq <- sum( values %in% value )  / length( values );
  obs.freq;
}

##########################################################################################
```

```
#
# Compute the stabilities of WALS features using the above estimation techniques
#
##########################################################################################

# Features above 138 don't work:
feature.selection <- 1:138
value.selection <- unlist ( sapply( feature.selection , function(x) { which( featValues[,"feat"] == x ) } ) )

# Get the statistics for all features, using 200 samples. The list 'results' is a list for all 653 values of the features 1 to 138:
results <- list()
for( i in value.selection )
{
  results[[i]] <- getcounts( featValues[i,1], featValues[i,2], 200 )
}

# Get the estimates for all results:
all.estimates <- list()
for( i in value.selection )
{
  all.estimates[[i]] <- estimates( results[[i]] )
}

# Extract the list of pAB transition probabilities:
pAB <- unlist( sapply( all.estimates, function(x){ x[3] } ) )

# Get the fraction of occurence of all values per feature: to be used in computing a weighted average of the stabilities of each value.
# This also nicely gets rid of the nasty problem of values that are only found in very few languages: Maslova's approach cannot estimate such groups.
# However, because such groups are small, they are getting a very low weight anyway now, so we don't have to worry about their influence:
proportion <- c()
for( i in feature.selection )
{
  t <- table(mat[,i])
  proportion <- c( proportion, t/sum(t) )
}

# Then we define the stability of a *value* as 1-pAB (i.e. the probability that a value to *not* change).
# To derive the stability of a *feature* we take the weighted average over these value-stabilities: frequent values count more than those with low frequencies:
stability <- xtabs( ( (1-pAB) * proportion ) ~ featValues[ value.selection, 1 ] )

# Save this list of feature stavilities to file for later use:
write.table( data.frame( "Feat"=as.character( names(stability) ), "Stability"=as.numeric(stability) ), "Maslova-stabilities.csv", sep="\t", quote=FALSE, row.names=FALSE );
```

References

1. Cysouw M (2011) Understanding transition probabilities. Linguistic Typology 15: 415-431.
2. Maslova E (2000) A dynamic approach to the verification of distributional universals. Linguistic Typology 4: 307–333.

3. Maslova E (2002) Distributional universals and the rate of type shifts: towards a dynamic approach to "probability sampling". URL `http://anothersumma.net/Publications/Sampling.pdf`. Lecture given at the 3rd Winter Typological School, Moscow. Accessed 2012 Dec 26.

4. Maslova E (2004) [Dynamics of typological distributions and stability of language types]. Voprosy Jazykoznanija 5: 3-16.

5. Maslova E, Nikitina T (2008) Stochastic universals and dynamics of cross-linguistic distributions: the case of alignment types. URL `http://www.anothersumma.net/Publications/ProbabilityPubl.html`. Accessed 2012 Dec 26.

6. Dediu D (2011) A bayesian phylogenetic approach to estimating the stability of linguistic features and the genetic biasing of tone. Proc R Soc B 278: 474-479.

7. Felsenstein J (2004) Inferring phylogenies. Sinauer Associates Inc.:Sunderland, Mass.

8. Ronquist F (2004) Bayesian inference of character evolution. Trends Ecol Evol 19: 475–481.

9. Haspelmath M, Dryer MS, Gil D, Comrie B, editors (2005) The World Atlas of Language Structures. Oxford University Press:UK.
